# Sexual Dimorphism in Language, and the Gender Shift Hypothesis of Homosexuality

**DOI:** 10.3389/fpsyg.2021.639887

**Published:** 2021-05-31

**Authors:** Severi Luoto

**Affiliations:** ^1^English, Drama and Writing Studies, The University of Auckland, Auckland, New Zealand; ^2^School of Psychology, The University of Auckland, Auckland, New Zealand

**Keywords:** sex differences, sexual orientation, psycholinguistics, personality, cognition, computerised text analysis, LIWC, evolutionary psychology

## Abstract

Psychological sex differences have been studied scientifically for more than a century, yet linguists still debate about the existence, magnitude, and causes of such differences in language use. Advances in psychology and cognitive neuroscience have shown the importance of sex and sexual orientation for various psychobehavioural traits, but the extent to which such differences manifest in language use is largely unexplored. Using computerised text analysis (Linguistic Inquiry and Word Count: LIWC 2015), this study found substantial psycholinguistic sexual dimorphism in a large corpus of English-language novels (*n* = 304) by heterosexual authors. The psycholinguistic sex differences largely aligned with known psychological sex differences, such as empathising–systemising, people–things orientation, and men’s more pronounced spatial cognitive styles and abilities. Furthermore, consistent with predictions from cognitive neuroscience, novels (*n* = 158) by lesbian authors showed minor signs of psycholinguistic masculinisation, while novels (*n* = 167) by homosexual men had a female-typical psycholinguistic pattern, supporting the gender shift hypothesis of homosexuality. The findings on this large corpus of 66.9 million words indicate how psychological group differences based on sex and sexual orientation manifest in language use in two centuries of literary art.

## Introduction

Psychological sex differences are perennially interesting to both scientists and laypeople. Psychological differences such as men’s higher systemising and women’s higher empathising, or men’s higher things orientation and women’s higher people orientation, have been reported in a variety of domains (Greenberg et al., 2018; Archer, 2019; Luoto, 2020b), with some psychologists arguing that the true extent of sex differences in human personality has been consistently underestimated (Del Giudice et al., 2012). Advances in cognitive neuroscience and evolutionary science have increased our knowledge of mammalian sexual differentiation of the brain and how this process creates sex differences and sexual orientation differences in various psychobehavioural traits in humans (Archer, 2019; Luoto et al., 2019a; Arnold, 2020; Luoto and Varella, 2021), but the way in which such differences may be reflected in language use is not well known. To broaden the current understanding of sex differences (Archer, 2019; Luoto and Varella, 2021) and sexual orientation differences (Xu et al., 2017; Luoto et al., 2019a; Luoto, 2020a), it is valuable to also broaden the material in which predictions from prevailing hypotheses on sex differences/similarities and sexual orientation differences/similarities are tested. In this article, those predictions are brought to bear on language use in literary art spanning more than 200 years.

One outcome of the sexual differentiation of the mammalian brain is variation in sexual orientation (Luoto et al., 2019a,b; Swift-Gallant et al., 2019; Bogaert and Skorska, 2020). Neurodevelopmental mechanisms underlying sexual orientation also lead to variation in a number of psychobehavioural traits (Xu et al., 2017; Luoto et al., 2019a; Luoto, 2020b). Therefore, sexual orientation tends to covary on a masculinity–femininity continuum with other psychobehavioural traits such as self-ascribed masculinity–femininity, occupational preferences, sociosexuality, personality traits, mental rotation, and verbal fluency (Rahman et al., 2003; Luoto et al., 2019a; Lippa, 2020; Xu et al., 2020) (though for some exceptions, e.g., bisexual women’s higher male-typicality relative to lesbian women on some psychobehavioural measures, see Luoto et al., 2019b; Luoto and Rantala, 2021). The biological mechanisms underlying variation in male (Bailey, 2018; Swift-Gallant, 2019; Swift-Gallant et al., 2019) and female sexual orientation (Luoto et al., 2019a,b) are becoming increasingly well understood, and such mechanisms also elucidate the existence of psychobehavioural sex differences in humans (Beltz et al., 2011; Berenbaum and Beltz, 2018; Archer, 2019; Arnold, 2020; Luoto, 2020a; Luoto and Varella, 2021).

Linguists, however, still debate about the magnitude of such differences in language use, and the study of sex differences and sexual orientation differences in language use has made very limited use of advances in other fields. One notable exception is a study that found sex differences and sexual orientation differences in verbal fluency, whereby homosexual individuals had shifted toward levels of fluency found in heterosexual members of the opposite sex (Rahman et al., 2003). Such findings support the gender shift hypothesis of homosexuality, which posits that homosexual men and women will be similar in certain neurobehavioural and psychological traits to their opposite-sex heterosexual counterparts (Bailey et al., 2016; Xu et al., 2017; Luoto et al., 2019a; Luoto, 2020a; Abé et al., 2021). However, not all language-based studies support the gender shift hypothesis: a study on personal ads found that homosexuals’ ads did not show the expected shift toward the opposite sex (Groom and Pennebaker, 2005). The 1,586 ads in that study were very short, only 171 words on average, providing a much weaker signal than novels which often comprise around 100,000 words each (in the current sample: *M* = 96,442 words per novel, *SD* = 52,838 words, total word count: 66.9 million). Thus, low statistical power may have explained the previous null results. Another small-scale study that compared 34 heterosexual men and 33 heterosexual women with 29 homosexual men and 29 homosexual women reported no significant differences in mean voice pitch between heterosexual and homosexual men or between heterosexual and homosexual women (Rendall et al., 2008). However, there were significant differences in the formant frequencies of vowels in homosexuals compared to their heterosexual counterparts (Rendall et al., 2008), lending some support for the gender shift hypothesis despite the low statistical power of the study.

The current study was designed to test whether known psychological sex differences and sexual orientation differences also manifest in language use. Personality researchers have highlighted the critical task of comparing self-reported personality with observer ratings and other, more objective evaluation methods (Del Giudice et al., 2012), making language use, particularly novels, a suitable complementary domain for cognitive and personality research (Tausczik and Pennebaker, 2010; Pennebaker and Ireland, 2011). While a great deal of research on gendered language is conducted by linguists, that research hardly ever seeks to understand linguistic findings in a broader context of *psychological* sex differences (e.g., Archer, 2019; Luoto and Varella, 2021), not to mention the predictions that can be made on sexual orientation differences in a cognitive neuroscience framework (Xu et al., 2017; Luoto et al., 2019a,b; Swift-Gallant et al., 2019). Fifteen predictions, summarised in Table 1 and presented in detail in Supplementary Table 1, were made based on relevant literature from psychology, linguistics, and cognitive neuroscience to guide this confirmatory research.

**TABLE 1:** A summary of predictions for this study and empirical support from the analyses.

LIWC category	Prediction in heterosexual authors	Empirical support for predicted sex difference	Empirical support for gender shift hypothesis
Analytical thinking	Men > women	Yes	Homosexual men
Conjunctions	Women > men	Limited, ns	No
Personal pronouns	Women > men	Yes	Homosexual men
Differentiation	Men > women	Opposite	Homosexual men
Cognitive words	Men > women	Opposite	Homosexual men
≥6 letters	Men > women	Limited, ns	No
Social words	Women > men	Yes	Homosexual men
Articles	Men > women	Yes	Homosexual men
Positive emotions	Women > men	Yes	Homosexual men
Negative emotions	Women > men	Limited, ns	Limited, ns
Sad	Women > men	Yes	Homosexual men
Anger	Men > women	Yes	Homosexual women
Anxiety	Women > men	Yes	Homosexual men
Death	Men > women	Yes	Homosexual men
Sexual words	Men > women	Limited, ns	Homosexual women
Present orientation	Men > women	No	No
Future orientation	Women > men	Yes	Limited, ns
Swear words	Men > women	Yes	Limited, ns
Numerical words	Men > women	Yes	Homosexual men
Work	Men > women	No	No
Risk	Men > women	No	No
Space	Men > women	Yes	Homosexual men

*Opposite = findings opposite to what was predicted. Ns, non-significant.*

Unlike most evolutionary scientists (e.g., Archer, 2019; Luoto and Varella, 2021), linguists tend to view sex differences in language use as caused by social stereotypes, researcher bias (Koolen, 2018, p. 137), or gender roles, with men being recorded more in workplace contexts and women more in domestic contexts (Baker, 2014). By “recording” males and females in largely similar contexts—at home, writing novels—the research design of this study can help evaluate whether these kinds of contextual factors may drive, or in this case eliminate, sex differences in language use. This study controls for contextual differences in language use because canonical and prize-winning male and female authors are presumed to write novels in similar settings in which they explore the contours of their creative minds: the contents of novels, by and large, reflect the products of authors’ imaginations, life experiences, and personal interests rather than what is present in their immediate environments.

As a fictional form of self-expression, novels provide a different kind of access into human minds than linguistic or psychological data collected using traditional psychological methods. Psychologists are able to use literary fiction as a point of entry into human minds (and linguists into human language use) that are far removed both in time and in space from the investigator. The authors of literature generally choose their own topics and go to great pains to select the words that best convey their stories, sentiments, and narrative visions. Consider, for example, Gustave Flaubert’s metacognitive reflection on the nature of writing literary fiction:

It is a delicious thing to write, to be no longer yourself but to move in an entire universe of your own creating. Today, for instance, as man and woman, both lover and mistress, I rode in a forest on an autumn afternoon under the yellow leaves, and I was also the horses, the leaves, the wind, the words my people uttered, even the red sun that made them almost close their love-drowned eyes (Flaubert, 1980).

Novels, therefore, are not strictly comparable with other linguistic corpora but offer an alternative, perhaps psychologically more accurate, or at least complementary, linguistic access point into human minds, enriching the ways in which psychological science is conducted whilst broadening its reach.

## Materials and Methods

### Materials

A power analysis was conducted to evaluate the required sample size for the study. It was initially decided that the sample size should be large enough to detect effect sizes of at least moderate magnitude (*d* > 0.35). The power analysis indicated that the sample size of each group of novels (male/female, heterosexual/bisexual/homosexual) should be 99 to be able to detect statistically significant effects of *d* > 0.36 with a power of 0.8 and *p* < 0.05 considered as a threshold for statistical significance. Thus, 99 novels was kept as a minimum target for each group of novels. As the data collection progressed, it became possible to acquire a larger sample of novels for each group, with the exception of bisexual novelists, who were more difficult to identify. The target sample size for each group was set as 150 novels with the intention of being able to detect group differences of approximately *d* > 0.29, which was a compromise between the availability of digitised novels and statistical power.

As shown in Supplementary Table 7, the heterosexual male sample comprised 151 novels with a total word count of 16.8 million words by 86 novelists. The heterosexual female sample comprised 153 novels with a total word count of 15.9 million words by 85 novelists. The homosexual male sample included 167 novels with a total word count of 15.7 million words by 55 novelists. The homosexual female sample included 158 novels with a total word count of 13 million words by 54 novelists. In addition, a sample of 65 novels (word count 5.5 million words) by 22 bisexual female authors was used when calculating estimated marginal means for the different groups of authors (see section “Estimated Marginal Means” below). For comparison, a corpus size of 200,000 words is deemed reasonably large for a discourse study (Friginal and Hardy, 2014, p. 220). Therefore, the samples collected for this study were very large by corpus linguistics standards. The total sample comprised 694 novels by 302 authors, totalling c. 66.9 million words. The details of the samples of novels are given in Supplementary Tables 2–6. The novels were written mainly by British, Irish, and North American authors. The novels included in the study were published mainly between 1800 and 2018 (see Supplementary Table 7 for descriptive statistics on the sample).^1^ The psycholinguistic data of the sample of novels used in this study are available in Luoto and van Cranenburgh (2021).

The novelists whose works were selected for this study were identified using literary anthologies (Kermode and Hollander, 1973; Gilbert and Gubar, 1985; Abrams, 1993; McCordick, 1996; Stavans and Acosta-Belén, 2011), biographical guides (Bloom, 1997; Griffin, 2003; Miller, 2006; Schmidt, 2014), and online lists of LGBT writers (Libraries, 2018; Wikipedia, 2018a,b). Additional novels were collected using literary awards to identify notable contemporary authors who may not yet be anthologised. Pulitzer Prize winners and National Book Award winners were added to the sample from 1965–2018 subject to availability of electronic versions of their novels. Booker Prize winners from 1969 to 2018 were also added to the sample. As the availability of novels authored by prize-winning authors was insufficient to reach the target sample size of 150 novels in each group of authors (male/female, heterosexual/bisexual/homosexual), the sample was broadened to include Booker prize and Pulitzer prize finalists (e.g., Lydia Millet, C. E. Morgan, and Lore Segal) to reach the target sample size.^2^ Canonical and prize-winning novelists were chosen partially because such works are more readily available in electronically readable form, partially because they represent culturally esteemed creative expressions of human existence, and partially because canonical books from the 19th and 20th centuries provide a larger temporal scope with which to test hypotheses, and thus to explore the extent to which psychological findings from contemporary populations replicate when analysing literary fiction written decades and centuries ago.

Since language use varies according to an individual’s age, with increasing age being characterised, for example, by more positive and fewer negative affect words, fewer self-references, more future-tense and fewer past-tense verbs, and increasing cognitive complexity (Pennebaker and Stone, 2003; Newman et al., 2008), age differences between authors—especially between different groups of authors compared in the analyses—may constitute a confounding variable for which adequate controls need to be introduced. Information on authors’ age at publication of each of their novels was recorded alongside the corpus collection and subsequently controlled for in the statistical analyses by including author’s age as a control variable in multilevel models. Pre-emptive elimination of age as a confounding variable was conducted by using a sampling protocol that focussed primarily on novels that each author wrote in their 30s and 40s. More specifically, the sampling protocol was designed so that two novels by each author were chosen for the sex difference analyses: one novel closest to author’s age of 35 at publication; the other closest to author’s age of 45 at publication (subject to availability). This protocol was also used with the prize-winning and finalist authors. Thus, the novels for which the literary awards were originally given were not always chosen for the analysis if, for instance, the prize-winning novel was published when the author was much older than 45 years. In such situations, another novel by the prize-winning or finalist author was chosen, subject to availability.

Authors’ sexual orientation was recorded using biographical information, including information on the sex of any partners (married or otherwise) that the authors had had or any self-identification related to sexual orientation that the authors may have made publicly known.^3^ Any author whose biographical information did not contain indications about their sexual orientation and/or behaviour were excluded from the study. Because of the difficulty of identifying a sample of homosexual and bisexual authors large enough to acquire the desired sample size of novels, more than two novels were collected from homosexual and bisexual authors when these were available. That way, the number of novels by homosexual authors reached a sample size similar to that of the heterosexual authors. Bisexual male authors were not included in the study because few authors could be identified as such. This is consistent with the generally low prevalence of bisexuality in men (Bailey et al., 2016).

Digitised versions of the novels were extracted from online databases, including Project Gutenberg, Project Gutenberg Australia, and Internet Archive,^4^ as well as two libraries. The novels extracted from Internet Archive had to be manually cleaned since they contained several copying errors and words that were hyphenated at line break. Linguistic Inquiry and Word Count (LIWC) does not recognise words hyphenated at line break, and so the results would have been inaccurate for the novels extracted from Internet Archive if the texts had not been manually cleaned. Novels collected from Project Gutenberg and Project Gutenberg Australia, as well as those collected from the two libraries, were subjected to a visual check for spelling errors and inappropriately hyphenated words, but no manual modifications were necessary in novels collected from these sources. Nevertheless, all novels were cleaned manually of prefaces, introductions, content tables, postscripts, biographical notes, author notes, footnotes, and publishers’ additional commercial material included at the end of many novels to prevent them from affecting the psycholinguistic analysis of the literary data (Luoto and van Cranenburgh, 2021).

The sample of novels by heterosexual authors included canonical works such as James Joyce’s *Ulysses*, Jane Austen’s *Sense and Sensibility*, and Herman Melville’s *Moby Dick*, as well as works by contemporary bestselling authors such as Ian McEwan and Kazuo Ishiguro. The homosexual samples included classics such as John Rechy’s *City of Night* from 1963 and Radclyffe Hall’s *The Well of Loneliness* from 1928 (see Supplementary Tables 2–6 for details of the novels). The homosexual and bisexual samples included many novels from authors who may be less well known: the sampling protocol for homosexual and bisexual authors was not based on literary prizewinners or finalists, because it was difficult (if not impossible) to obtain samples that were large enough that way.

### Methods

#### Linguistic Inquiry and Word Count (LIWC)

A suitable research design for a large-scale corpus analysis includes the greatest degree of automatisation that is possible without jeopardising the integrity of the results. LIWC was deemed the most appropriate research tool as it provides insight into a range of psychological processes and individual differences in language use (Tausczik and Pennebaker, 2010; Lanning et al., 2018). Tracking language use with psycholinguistic tools such as LIWC is similar to tracking a person’s gaze: it gives us natural clues on where people’s attention is focussed (Tausczik and Pennebaker, 2010).

Linguistic Inquiry and Word Count has an in-built English dictionary which categorises text into approximately 90 psycholinguistic output variables (Pennebaker et al., 2015). For each text file, LIWC reads one word at a time and compares it with the in-built dictionary file, creating an output which shows the relative frequency of words tagged for each psycholinguistic variable. Each of the output variables is written as one column of data to an output file; each text file is written as a row. The data output in columns includes the file name and word count, four summary language variables (analytical thinking, clout, authenticity, and emotional tone), three general descriptor categories (words per sentence, percent of target words captured by the dictionary, and percent of words in the text that are longer than six letters), 21 standard linguistic dimensions (e.g., percentage of pronouns, articles, and verbs), 41 psychological construct categories (e.g., affect, cognition, biological processes, and drives), six personal concern categories (e.g., work, home, and leisure activities), five informal language markers (assents, fillers, swear words, netspeak, and non-fluencies), and 12 punctuation categories (e.g., periods, commas, and semicolons) (Pennebaker et al., 2015). The four summary variables (analytical thinking, clout, authenticity, and emotional tone) are the only non-transparent dimensions in the LIWC2015 output: all the other LIWC variables are a percentage of total words in each category per text (Luoto and van Cranenburgh, 2021). Table 1 shows a summary of the 24 LIWC categories and predictions; for details on the predictions and the 24 LIWC categories used in the analyses, see the Supplementary Materials.

#### Statistical Analyses

As indicated in the preregistration, available at https://aspredicted.org/5hg8z.pdf, independent samples *t*-tests were used in SPSS version 26 to compare the means of each variable between different groups of authors (Supplementary Table 9). These tests were conducted to determine whether there is statistical evidence showing that the means are significantly different between novels written by authors in the different sex/sexual orientation groups.

Although multilevel analysis was not included in the preregistration, the need to use multilevel modelling became apparent during the data collection when more than one novel was collected for most of the authors included in the study. Multiple novels from one author had to be included because of the difficulty, first, of identifying suitable novelists (finding a large sample of lesbian authors, for example, turned out somewhat challenging), and second, lack of availability of novels in electronically readable form. The fact that more than one novel was often included by authors leads to data that are “clustered” within a study subject: observations from the same study subject are likely to be more highly correlated with one another than with observations from another participant. Thus, the total sample size does not provide an accurate reflection of the information/level-of-evidence in the data (Moen et al., 2016). The data will be incorrectly analysed if the correlation of observations from the same participant is ignored and each such observation is treated as an independent observation. *Sex* was a fixed variable with fixed effects in this study, while *author* was a random variable with random effects. All multilevel models and estimated marginal means controlled for potential confounds arising from differences in publication year and authors’ age between the samples (see Supplementary Tables 8, 10, 11 and Figures 4–6). Author’s country was not included as a separate level in the multilevel model because of the low number of countries included in the sample. The majority of the authors in this study were either American, British, Irish, or Canadian (see Supplementary Tables 2–6 for details on the novels and authors used in this study).

Mahalanobis *D*s were calculated using multilevel univariate *d*s (acquired with SPSS version 26, see Supplementary Tables 8, 10, 11) controlling for publication year and author’s age at publication. Mahalanobis *D* calculations were done using the supplementary material provided in Del Giudice (2019). Estimated marginal means (see section “Estimated Marginal Means”) were calculated using a multilevel model which adjusts the means based on variation in publication year and author’s age at publication. There is no consensus among statisticians on which effect size to use in multilevel modelling (Garson, 2014). Some authors have suggested that the unstandardised beta can be used to calculate *d* in multilevel models using pooled standard deviation and the following formula: *d* = *b/SD*_*pooled*_ (Baguley, 2009; Feingold, 2015). This protocol was followed in this study.

Alpha level adjustments were not performed because the tests reported in this study were conducted on different null hypotheses. Alpha adjustment is only necessary when different tests are conducted on the same null hypothesis (Rubin, 2017). The different predictions tested here pertained to differences in each psycholinguistic domain separately rather than testing the simple *general* hypothesis of the existence of sex differences and/or sexual orientation differences. Because the analyses were aimed at testing specific predictions that pertain to different psycholinguistic traits, each prediction can be either supported or not supported by the data, and therefore it is not necessary to adjust alpha value for multiple comparisons (cf. Rubin, 2017).

## Results

### Sex Differences

**FIGURE 1:**
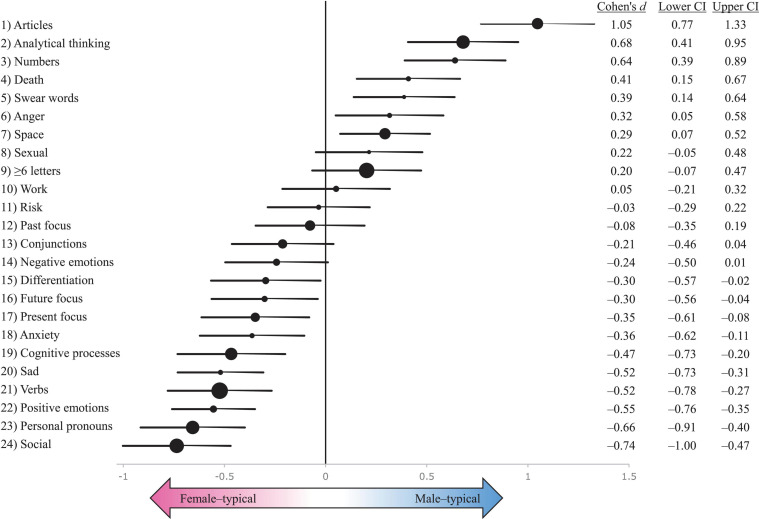
A summary of sex difference effect sizes (Cohen’s *d*s) with 95% confidence intervals (CI) on 24 psycholinguistic variables. The sample comprised 304 novels by 86 heterosexual male novelists and 85 heterosexual female novelists, published between 1800 and 2018. Cohen’s *d*s for each psycholinguistic variable are depicted as filled circles, which are scaled to reflect the prevalence of each word category in the overall sample: the larger the circle, the more frequently do words in that category occur in the overall sample. Positive *d*s represent male advantage; negative *d*s indicate female advantage. Psycholinguistic categories that are used more frequently by male authors appear on the right side of the figure, and psycholinguistic categories favoured by female authors appear on the left side of the figure. Error bars represent 95% confidence intervals. The effect sizes and CIs are calculated based on a multilevel model which controls for variation in publication year and author’s age at publication (Supplementary Table 8).

Figure 1 summarises the sex difference effect sizes from a multilevel model which included sex as a fixed variable with fixed effects, author as a random variable with random effects, and authors’ age and publication year as covariates (Supplementary Table 8).

#### Articles, Social Words, and Personal Pronouns

In line with prior psychological and linguistic research, heterosexual female authors showed a substantially higher focus on people than heterosexual male authors. Female authors used significantly more personal pronouns (*d* = −0.66) and social words (*d* = −0.74) than male authors, while male authors used significantly more articles (*d* = 1.05) than female authors. To the extent that article frequency indexes higher focus on objects (rather than on people) (Newman et al., 2008), male authors’ language use reflected a higher salience of objects, which would also be consistent with findings on psychological sex differences in things and people orientation (Archer, 2019; Luoto, 2020b) as well as with the lower frequency of social words and personal pronouns in male authors’ language. These effect sizes are much larger than previously reported when Linguistic Inquiry and Word Count (LIWC) has been used to analyse sex differences in language use (Newman et al., 2008), possibly because this study introduced stricter controls (domain of writing, publication year, authors’ age, authors’ sexual orientation) than prior studies have (Newman et al., 2008). This suggests that prior estimates about the magnitude of sex differences in language use (Newman et al., 2008) may have been underestimated.

#### Emotion Words

Consistent with prior research and with predictions (Supplementary Table 1), female authors used language that was significantly more emotionally laden than male authors’ language. This was true with both positive and negative emotion words, with the exception of anger-related words, which were used more frequently by male authors (Figure 1), as predicted.

#### Analytical Thinking

Heterosexual male authors were predicted to score slightly higher than heterosexual female authors on the factor-analytically derived composite score ‘analytical thinking,’ calculated by LIWC based on eight function word categories (Pennebaker et al., 2014). Male authors had a substantially higher analytical thinking score than female authors, resulting in a large effect size of *d* = 0.68. This finding is consistent with the hypothesis that psychological sex differences in systemising–empathising (Greenberg et al., 2018; Archer, 2019) are also reflected in language use. The effect size of *d* = 0.68 for analytical thinking falls within the typical range of sex difference effect sizes reported for systemising, with Cohen’s *d*s ranging between 0.31 and 1.21 (Greenberg et al., 2018; Archer, 2019), although analytical thinking as measured by LIWC and systemising quotient as measured in psychological research (e.g., Greenberg et al., 2018) may not be exactly equivalent psychological constructs.

#### Cognitive Processes and Cognitive Complexity

Male authors were expected (Supplementary Table 1) to use more differentiation words, cognitive words, and words with six or more letters—LIWC categories which have been previously associated with cognitive complexity (Tausczik and Pennebaker, 2010). There were no significant sex differences in the use of words longer than six letters or conjunctions, although the small differences that did exist (*d* = 0.20 and *d* = −0.20, respectively) were both in the predicted direction.

Female authors had significantly higher frequencies of words related to cognitive processes (insight, causality, discrepancies, tentativeness, certainty, and differentiation). Female authors’ higher use of cognitive process words could, in part, reflect psychological sex differences in theory of mind, whereby women are better than men at interpreting others’ intentions and actions, demonstrating an improved domain-specific ability to read others’ minds (Ibanez et al., 2013). Psychologically, this sex difference is mediated by empathy (Ibanez et al., 2013) for which sex differences are well known (Archer, 2019); developmentally, theory of mind is affected by prenatal androgen exposure (Khorashad et al., 2018), which is an important neurodevelopmental mechanism giving rise to many psychobehavioural sex differences and sexual orientation differences (Luoto et al., 2019a; Arnold, 2020; Bogaert and Skorska, 2020; Luoto and Varella, 2021). The sex difference in cognitive process words could therefore be mediated by women’s higher focus on people and on emotions. To test this hypothesis, I ran a *post hoc* analysis with social words, positive emotion words, anxiety words, and sadness-related words added as covariates in the multilevel model on sex differences in cognitive process words (Supplementary Text). These *post hoc* analyses indicated that female authors’ higher cognitive process ratings pivot on the heightened psycholinguistic salience of people and emotions for women (Supplementary Text), which is consistent with known psychological sex differences in emotionality and people orientation (Archer, 2019; Luoto, 2020b). Female authors’ higher psycholinguistic salience of people and emotions also reduces female authors’ analytical thinking scores relative to male authors (Supplementary Text). In other words, on average, female authors appear to show more complex cognitive processes about emotions and people, which reduces female authors’ analytical thinking (as measured by LIWC).

#### Numerical Words

Male authors used significantly more numbers and numerical words than female authors (Figure 1). This psycholinguistic finding may reflect the sex difference whereby mathematics is much more likely to be an academic strength for boys than it is for girls (Stoet and Geary, 2018; Eriksson et al., 2020) and whereby women may be intrinsically less interested in mathematics-intensive subjects than men (Thelwall et al., 2019; Luoto, 2020b).

#### Spatial Words

Male authors used more spatial words than female authors (*d* = 0.29), consistent with predictions (Supplementary Table 1) as well as with other research in cognitive science, which has reported better mental rotation skills (*d* = 0.66), visuospatial abilities (*d* = 0.48), and spatial visualisation (*d* = 0.23) in men relative to women (Archer, 2019).

#### Swearing and Sexual Words

Swearing showed an intermediate sex difference in the predicted direction: male authors used swear words significantly more than female authors (Figure 1). Expected sex differences in sexual words were not fully borne out by the data. The frequency of sexual words was almost equally low in male-authored and female-authored novels (0.13% and 0.11%, respectively, Supplementary Table 9). The sex difference effect size was non-trivial though statistically non-significant (*d* = 0.22, *p* = 0.109, Supplementary Table 8), and a larger sample size would be needed to reliably detect effects of this magnitude. Since the current finding is aligned with the theoretical prediction (Supplementary Table 1), it does provide tentative support to the hypothesised higher salience of sexual motivation in male authors, which remains to be confirmed in future research. Nevertheless, the relatively low frequency of sexual words in canonical and prize-winning novelists’ works may also represent a psycholinguistic floor effect driven by the literary prestige of the sampled texts.

#### Death

Relative to female authors, male authors employed language that had a significantly higher frequency of words related to death (Figure 1). Psychological research has not found any significant sex differences in mortality salience (Burke et al., 2010). However, there is a substantially greater likelihood for males to suffer death from external causes at a young age (Gottschall, 2008; Archer, 2019), while sex differences in longevity, favouring the homogametic sex, are well-known across countries, time periods, and even species (Austad, 2006; Austad and Fischer, 2016; Xirocostas et al., 2020). These factors, including significantly higher male mortality in wars throughout the study period, may partially account for male authors’ higher use of death-related words.

#### Work and Risk

Contrary to the prediction, there was no sex difference in work-related words or risk-related words (Figure 1 and Supplementary Table 8). Although there is a moderate behavioural sex difference in risk-taking in general (Archer, 2019; Luoto and Varella, 2021), this was not reflected in the language used in the novels. A potential explanation for this null finding is that even though men take more risks, women, by virtue of their higher neuroticism, anxiety, and harm avoidance (Archer, 2019), could *perceive* more risks in their environment than men (Luoto and Varella, 2021), thus attenuating any sex differences in manifest risk-related language use in novels.

#### Time Orientation and Verbs

Female authors’ word use showed a higher present focus as well as future focus than male authors’ word use (Figure 1 and Supplementary Table 8). Female authors’ higher focus on the present was contrary to predictions but consistent with prior research (Newman et al., 2008). Female authors’ higher focus on the future was consistent with predictions. Past focus showed only a small, non-significant sex difference (Figure 1). Since verb conjugations are a key indicator of past/present/future focus, higher absolute verb use by female authors could have spuriously caused a part of the sex differences in present focus and future focus. I therefore ran a further *post hoc* analysis on verb use. Although sex differences in verb frequency have been reported in prior research (Johannsen et al., 2015; Rybicki, 2016), sex differences in verb use were not included in the list of predictions (Supplementary Table 1) because existing psychological theory does not clearly lead to any predictions on such differences. Nevertheless, female authors had a substantially higher use of verbs than male authors (Figure 1). Additional analyses (Supplementary Materials) revealed that female authors’ higher focus on present and future was indeed almost fully driven by female authors’ higher verb use.

### Sexual Orientation Differences in Males

**FIGURE 2:**
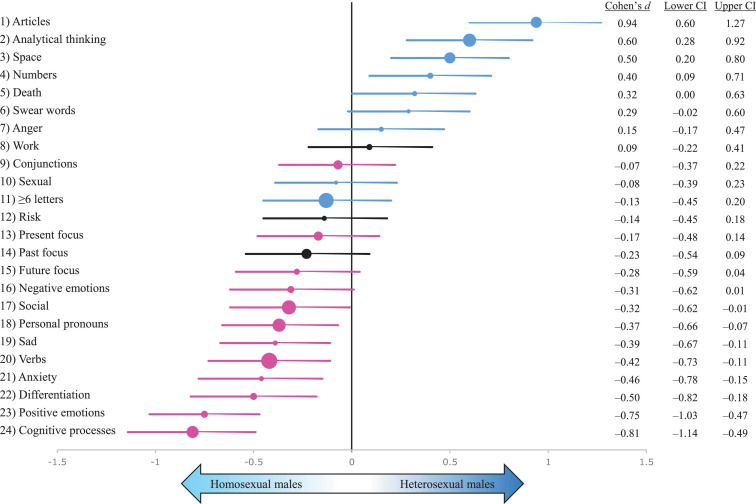
A summary of multilevel sexual orientation effect sizes (Cohen’s *d*s) with 95% confidence intervals (CI) on 24 psycholinguistic variables. The sample comprised 318 novels by 86 heterosexual male novelists (*n* = 151 novels) and 55 homosexual male novelists (*n* = 167 novels). Cohen’s *d*s for each psycholinguistic variable are depicted as filled circles, which are scaled to reflect the prevalence of each word category in the sample: the larger the circle, the more frequently do words in that category occur in the sample. Positive *d*s represent higher scores in heterosexual male authors’ novels; negative *d*s indicate higher scores in homosexual male authors’ novels. Error bars represent 95% confidence intervals. The effect sizes and CIs presented here are calculated based on a multilevel model which includes publication year and author’s age at publication as covariates. The symbols are colour-coded based on the sex difference results (Supplementary Table 8) so that blue = male-typical; magenta = female-typical; black = no sex difference.

Figure 2 summarises the male sexual orientation (homosexual/heterosexual) effect sizes from a multilevel model which included sexual orientation as a fixed variable with fixed effects, author as a random variable with random effects, and authors’ age and publication year as covariates (Supplementary Table 10).

Homosexual male authors showed a clear pattern of psycholinguistic feminisation (Figure 2). The male-typical traits (blue symbols, Figure 2) cluster in the upper right-hand corner, which reflects the fact that homosexual male authors tended to score low on these male-typical traits. The female-typical traits (magenta symbols, Figure 2) cluster in the lower left-hand side of the figure. This reflects the high scores that homosexual male authors had on those traits. The three traits—work-related words, risk-related words, and past focus—that did not show any clear sex differences in Figure 1 (marked as black symbols in Figure 2) cluster in the middle of Figure 2, showing no significant differences between males differing in sexual orientation.^5^ Overall, the results demonstrate that homosexual male authors’ psycholinguistic profiles were highly feminised on most of the psycholinguistic variables (Figure 2).

### Sexual Orientation Differences in Females

**FIGURE 3:**
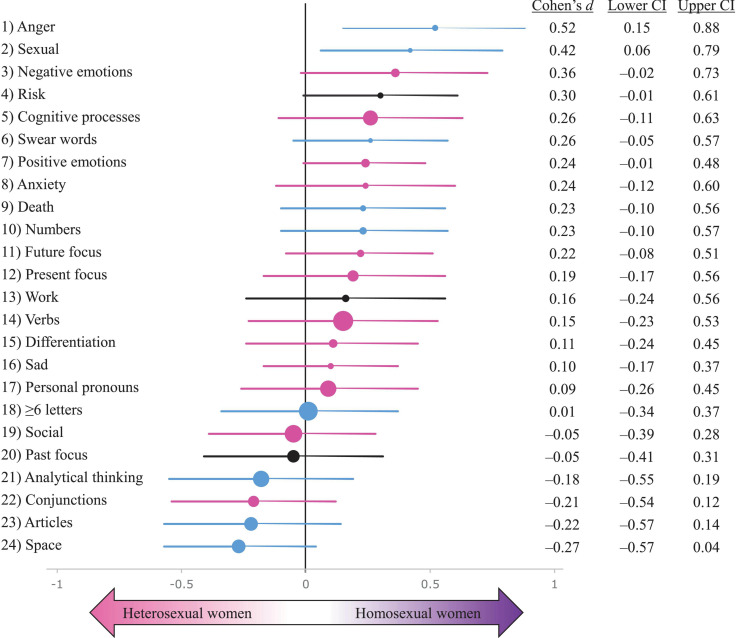
A summary of multilevel sexual orientation effect sizes (Cohen’s *d*s) with 95% confidence intervals (CI) on 24 psycholinguistic variables. The sample comprised 311 novels by 85 heterosexual female novelists (*n* = 153 novels) and 54 homosexual female novelists (*n* = 158 novels). Cohen’s *d*s for each psycholinguistic variable are depicted as filled circles, which are scaled to reflect the prevalence of each word category in the sample: the larger the circle, the more frequently do words in that category occur in the sample. Positive *d*s represent higher scores in homosexual female authors’ novels; negative *d*s indicate higher scores in heterosexual female authors’ novels. Error bars represent 95% confidence intervals. The effect sizes and CIs presented here are calculated based on a multilevel model which includes publication year and author’s age at publication as covariates. The symbols are colour-coded based on the sex difference results (Supplementary Table 8) so that blue = male-typical; magenta = female-typical; black = no sex difference.

Figure 3 summarises the female sexual orientation (homosexual/heterosexual) effect sizes from a multilevel model which included sexual orientation as a fixed variable with fixed effects, author as a random variable with random effects, and authors’ age and publication year as covariates (Supplementary Table 11).

Only three psycholinguistic categories showed statistically significant differences between female authors differing in sexual orientation (Figure 3 and Supplementary Table 11). Lesbian authors’ novels had a hyperfeminine pattern of a higher frequency of positive emotion words (*d* = 0.24) relative to heterosexual female authors’ novels. Though the effect was small and only marginally significant (Supplementary Table 11), this finding contradicted the prediction of psycholinguistic masculinisation in lesbian authors (Supplementary Table 1). Anger-related words and sexual words indicated a significantly masculinised pattern in novels by lesbian authors (Figure 3).

### Summary

Table 1 collates the predictions and the empirical support provided for them in these samples of novels.

### Mahalanobis *D*: Multivariate Effect Sizes

I calculated estimates of multivariate effect size Mahalanobis *D*s (Del Giudice, 2013, 2019) to further analyse the magnitude of sex differences and sexual orientation differences in this sample. To reduce bias in the calculations, it is recommended that the number of variables which Mahalanobis *D* calculations are based on are relative to the sample size: Del Giudice (2013) recommends having at least 100 cases per variable as a reasonable rule of thumb in most research contexts. Most of the LIWC categories analysed in this study have some overlap between one another: analytical thinking overlaps with articles and conjunctions; the negative emotions category overlaps with anger, anxiety, and sadness; verbs overlap with several categories, as do sexual words and social words. Therefore, it may be problematic to make Mahalanobis *D* calculations on univariate *d*s based on all of the 24 categories used in this study, as there can be some overlap between many of the categories. To avoid this problem, Mahalanobis *D* calculations were based on three basic non-overlapping linguistic LIWC categories: articles, personal pronouns, and numbers (including numerical words).

Using only three LIWC variables—articles, personal pronouns, and numbers—the multivariate effect size for sex differences was *D* = 1.13 (while controlling for variation in publication year and authors’ age at publication). A multivariate effect size of *D* = 1.13 means that there is only a 57% overlap between the heterosexual male and female distributions, signifying a very large multivariate difference between the groups. A multivariate effect size of this magnitude yields a probability of correct classification (by sex) of about 72% (Del Giudice, 2019).

Calculating Mahalanobis *D* for the multivariate difference between homosexual male and heterosexual male samples using the same three variables—articles, personal pronouns, and numbers—results in *D* = 1.02. This very large multivariate difference means that the overlap between the samples in the multivariate space of these three variables is 61%. The probability of correct classification (by sexual orientation) is about 69%, meaning that, using these three variables alone, homosexual men are psycholinguistically almost as distinguishable from heterosexual men as heterosexual women are.

Using these three variables yields Mahalanobis *D* = 0.34 when comparing the homosexual and heterosexual female samples. This relatively small multivariate difference between heterosexual and homosexual female authors indicates that the overlap between the samples in the multivariate space of these three variables is relatively large, 86.5%. The probability of correct classification is approximately 57%. It would therefore be relatively unreliable to distinguish lesbian authors from heterosexual female authors based on these three variables.

### Estimated Marginal Means

**FIGURE 4:**
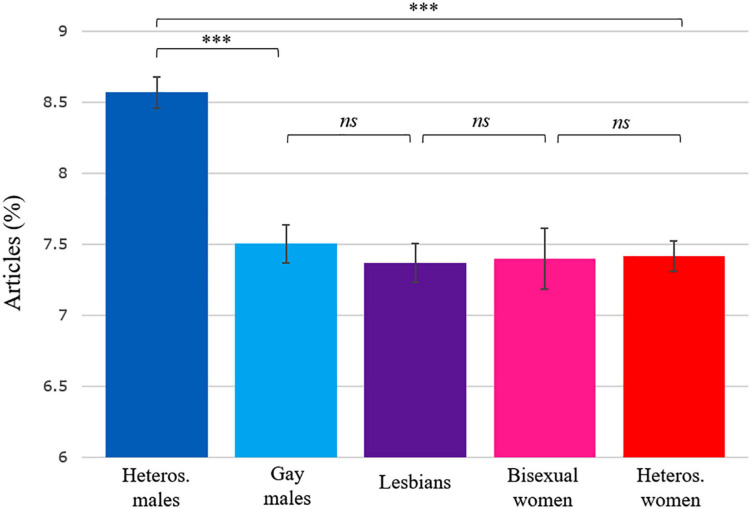
Estimated marginal means and standard errors for article frequency in each group of authors. The estimated marginal means are adjusted for differences in publication year and author’s age at publication. ^∗∗∗^*p* < 0.001. *ns*, non-significant.

**FIGURE 5:**
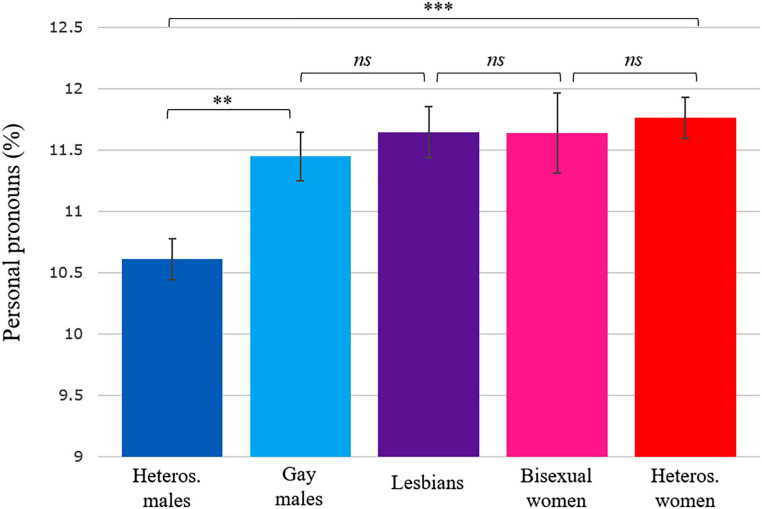
Estimated marginal means and standard errors for personal pronoun frequency in each group of authors. The estimated marginal means are adjusted for differences in publication year and author’s age at publication. ^∗∗∗^*p* < 0.001, ^∗∗^*p* < 0.01. *ns*, non-significant.

**FIGURE 6:**
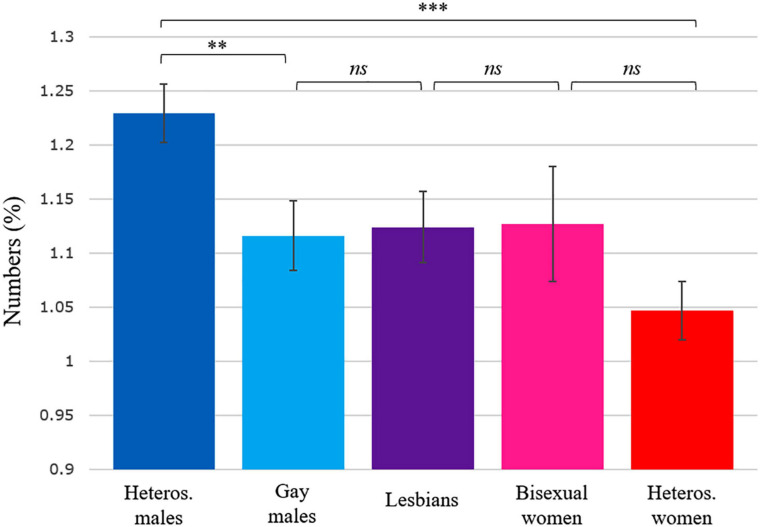
Estimated marginal means and standard errors for frequency of numbers and numerical words in each group of authors. The estimated marginal means are adjusted for differences in publication year and author’s age at publication. ***p < 0.001, **p < 0.01. *ns*, non-significant.

Figures 4–6 show estimated marginal means for articles, personal pronouns, and numbers that are adjusted for differences in publication year and author’s age at publication. The figures include pairwise comparisons between five groups of authors differing in sex and sexual orientation. Besides the four samples discussed above, a smaller sample of novels by bisexual female authors (*n* = 65 novels, totalling 5.5 million words by 22 novelists) was also added to these analyses to show how bisexual female authors may differ from the other groups of authors. The three Figures 4–6 indicate that the only statistically significant differences on these three variables occurred between heterosexual male authors and the four other groups.

## Discussion

A corpus of 694 novels comprising 66.9 million words spanning more than two centuries of literary art was compiled to determine the extent to which heterosexual male and female authors, and homosexual male and female authors as well as a small sample of bisexual female authors, produced psycholinguistic outputs that differed in predictable ways. The results indicated significant sexual dimorphism^6^ in the language used in literary fiction written by heterosexual male and female authors, consistent with predictions based on cognitive neuroscience, psychology, and evolutionary science, while also providing support for the gender shift hypothesis of homosexuality (Abé et al., 2021; Luoto et al., 2019a; Luoto, 2020a). The gender shift hypothesis of homosexuality was strongly supported in homosexual males—who produced female-typical psycholinguistic outputs—whereas the evidence among homosexual female authors was substantially weaker, as they showed only a minor psycholinguistic shift in the heterosexual male direction.

While writers and readers, and speakers and listeners, have long been interested in how men and women may use language in slightly-to-vastly different ways, this study helps to clarify the existence, magnitude, and possible psychological underpinnings of sex differences in language use, which appear in areas over which writers would not be exercising sex-conscious psycholinguistic control. It would be difficult to conceive, for instance, how male authors might consciously increase the frequency with which they use articles (‘a,’ ‘an,’ and ‘the’) because they associate such language use with some nebulously “desirable” characteristics related to their ideas of “masculinity.” It is difficult, in other words, to explain the findings with the social role theory of gender roles, which would further struggle to provide a plausible explanation for homosexual male authors’ female-typical language use. If homosexual males were socialised into the male gender role, why do they use language in a way that resembles heterosexual women’s language use? To the extent that these findings represent non-conscious, natural ways of using language, they also suggest that homosexuality is not a conscious choice (Luoto et al., 2019a; Swift-Gallant et al., 2019; Bogaert and Skorska, 2020). It is highly unlikely, after all, that homosexual male authors have consciously chosen to write in a more female-typical way, of which they could have had limited notion at the level of psycholinguistic minutiae.

While some people argue that socialisation into gender roles underlies sex differences in humans, this hypothesis becomes implausible when considering the biological, developmental, neuroscientific, and cross-cultural evidence more broadly (Christov-Moore et al., 2014; Schmitt, 2015; Janicke et al., 2016; Archer, 2019; Del Giudice, 2019; Luoto et al., 2019a; Atari et al., 2020; Stoet and Geary, 2020; Luoto and Varella, 2021). Most sex differences in personality are of a higher magnitude in more gender-egalitarian countries than in less gender-egalitarian countries, which is the opposite of what the gender role hypothesis would predict (Schmitt et al., 2008; Falk and Hermle, 2018; Atari et al., 2020; Stoet and Geary, 2020). Furthermore, since evolutionary processes pre-date social conceptualisations of gender roles by millions of years, a full explanation of socialisation into gender roles and the effects it has on sexually differentiated traits and behaviours would need to account for how evolutionary processes act as precursors to gender roles (Janicke et al., 2016; Archer, 2019; Luoto and Varella, 2021; Luoto et al., 2021).

**FIGURE 7:**
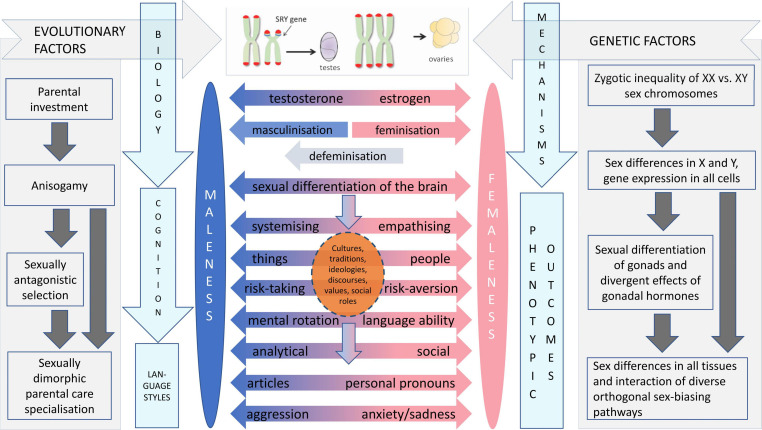
The evolutionary–developmental origins and proximate mechanisms underlying psychobehavioural sex differences, including those in language use. Figure adapted from Luoto and Varella (2021).

Ultimately, psychobehavioural sex differences arise from sexual selection, sexual differentiation of the mammalian brain, sexual division of labor, and their interactions (Figure 7) (Luoto and Varella, 2021). Sexual selection and sex differences in parental investment have exerted and currently exert selection pressures on status-striving and power-seeking among men more than in women (Luoto, 2019), contributing to men’s higher competition, aggression, risk-taking, sociosexuality, and men taking on more leadership positions than women, particularly at higher organisational and societal levels (Luoto and Varella, 2021). Sex differences in parental investment and mating competition coevolve with parental care specialisation, which can partially contribute to such psychobehavioural sex differences as found in empathising, people orientation, risk-taking, neuroticism, mate choice, sociosexuality, aggression, violence, leadership, and dominance (Archer, 2019; Henshaw et al., 2019; Luoto et al., 2019a; Luoto and Varella, 2021). Sexually dimorphic ultimate evolutionary functions exert an influence on psychobehavioural sex differences via various biological mechanisms, leading to sexually dimorphic language use which, further down the evolutionary–developmental trajectory, also reflects other known psychobehavioural sex differences (Figure 7).

Comparative research provides further evidence against social role theories of human sex differences. Evidence of sex-biassed treatment by others (equivalent to what proponents of social constructionist hypotheses think of as socialisation into gender roles in humans) is lacking in non-human animals. Behaviours of mothers toward female and male offspring show little to no difference in the few species that have been studied (Lonsdorf, 2017), yet such species show sex differences in behavioural, physical, and social development that resemble those found in infant humans (Christov-Moore et al., 2014; Lonsdorf, 2017; Archer, 2019). These include sex differences in species-typical behaviours such as grooming, playing, object manipulation, and extractive foraging (Lonsdorf, 2017). Immature chimpanzee males, for instance, engaged in more object-oriented play than females (Koops et al., 2015). Under 5-week-old newborn rhesus macaque females that were raised in a controlled postnatal environment looked more at computer-generated faces of other rhesus macaques and engaged in more affiliative behaviour with a human caregiver than newborn rhesus macaque males did (Simpson et al., 2016). Similar findings have been reported in humans: 12-month-old female infants showed a higher relative preference for a moving face over a moving car than males did (*d* = −0.64) (Lutchmaya and Baron-Cohen, 2002). In humans, vervet monkeys, and rhesus macaques, females have been observed playing longer with dolls and plush toys, while males play longer with wheeled toys (Christov-Moore et al., 2014). Asian elephant females have a tendency to be more social and gregarious than males (Seltmann et al., 2019). In humans and non-human primates, females engage in social grooming more often than males (Lonsdorf, 2017). In hamsters and humans, females find same-sex social interactions more rewarding than males do. Oxytocin plays a similar mechanistic role in social reward processing in a number of species, suggesting that sociality and sex differences in sociality may arise from a common evolutionary origin (Feng et al., 2015; Hung et al., 2017; Borland et al., 2018).

Furthermore, evolutionarily conserved hormonal mechanisms, such as testosterone, are associated with language use and other sexually dimorphic phenotypes (Hoskin and Meldrum, 2018; Mascaro et al., 2018; Archer, 2019; Luoto et al., 2019a), providing a biological basis for the emergence of sexually differentiated traits. Many lines of research, including longitudinal research in humans, support this theory. While hormone exposure significantly predicted gender development in girls, mothers’ socialisation to feminise the daughters had negligible effects: women exposed to more testosterone in prenatal development showed masculinised behaviours in adulthood despite parents’ socialisation efforts to have the daughters behave in a more feminine way (Udry, 2000).

Evidence for the relationship between testosterone and many sexually dimorphic phenotypes spans several different areas of research (Björkqvist, 2018; Hoskin and Meldrum, 2018; Luoto et al., 2019a; Muñoz-Reyes et al., 2020). It is noteworthy that psychological research has not found reliably occurring differences in anger frequency; instead, sex differences have been found in verbal and physical aggression, both being higher in men (Archer, 2019). Thus, the slightly higher frequency of anger-related words in male authors’ novels (*d* = 0.32, Figure 1) does have some equivalents in psychological research. The use of anger-related words is positively correlated with circulating testosterone levels and with polymorphisms in the androgen receptor gene (Mascaro et al., 2018), which make cells more susceptible to the masculinising influence of testosterone. These findings indicate the existence of a plausible biological mechanism (Geniole et al., 2019; Luoto et al., 2019a) which creates sex differences in anger-related language use as well as other psychobehavioural sex differences, including people–things orientation, risk-taking, and theory of mind (Khorashad et al., 2018; Luoto, 2020b; Vaskinn et al., 2020; Luoto and Varella, 2021). Furthermore, the finding of higher anger-related words and sexual words in lesbian authors relative to heterosexual women is consistent with existing findings on psychobehavioural masculinisation in non-heterosexual women, including higher sociosexuality, sensation-seeking, psychopathy, and incarceration rates compared with heterosexual women (Luoto et al., 2019a,b) (though see Gil-Llario et al., 2015 who reported lower sexual sensation seeking in self-identified lesbians than in heterosexual women).

An important contribution of this study was the ability to predict and explain sexual dimorphism in language using psychology and cognitive neuroscience. A related major result is that prior research on sex differences and sexual orientation differences in these fields have clear equivalents in the psycholinguistic outputs of authors writing literary fiction decades and centuries ago, suggesting that psychological sex differences may be relatively stable across time and across different domains—that is, they manifest not only via questionnaires, psychological tests, and behavioural measures, but also in the artistic and linguistic forms of imaginary self-expression enabled by literary fiction; and they manifest not only in contemporary population-based samples, but also in the highly specialised sample of writers of canonical literary fiction from decades and centuries ago. This coherence across different areas of research and across different time periods allays concerns that could be raised about the generalisability of the current findings.

### Limitations

A clear limitation of this study was that the analyses were conducted only on English-language material. Future studies are therefore encouraged in other languages to provide an estimate of the generalisability of these findings across other languages. Corresponding results have, however, been reported in a number of languages using various literary and non-literary sources, though few studies have distinguished between writers of different sexual orientations (cf. Argamon et al., 2009; Johannsen et al., 2015; Chen et al., 2018; Koolen, 2018).

Another potential limitation of this study is that effect sizes can become biassed because of range restriction, which refers to a process in which the participants of a study are, directly or indirectly, selected from the original population on the basis of their personal characteristics (Del Giudice, 2019). In the current case, all samples of novels are likely to suffer from range restrictions as the novels were not sampled at random from all novels ever written by heterosexual or homosexual men and women; rather, canonical and prizewinning novels were mostly used, although the non-heterosexual samples also included less well-known novels because of the necessity to reach a large enough sample size. What is more, it may not be possible to directly extrapolate these findings on novelists to the respective groups of *all* lesbian women or *all* gay men or *all* heterosexual women and men. That is because only a small subset of each of these groups is likely to write and publish novels, particularly novels that reach a canonical status; thus, the sampling of such individuals may not be generalisable to the full sample of non-novelists in each group. This limitation can be addressed by comparing the present findings with existing findings on similar group differences that have been acquired using other kinds of methodologies and sampling protocols on non-novelists. Thus, to the extent that the current findings are consistent with the findings of other sex difference and sexual orientation difference studies (which they generally tended to be), the sampling problem of focussing only on novelists is mitigated.

This study was also limited in the sense that the heterosexual sample was drawn from canonical and prize-winning authors’ works: these culturally esteemed works may not generalise to the other 99% of literature ever written (Moretti, 2005, 2013). Furthermore, as most of the non-heterosexual sample comprised works that were not canonical nor prize-winning (necessarily so because of the difficulty of obtaining such samples that would have been large enough for adequate statistical power), I cannot rule out the possibility that the psycholinguistic differences observed in this study between authors of different sexual orientation could have been partially driven by the differences in canonicity and/or literary prestige between the samples. Nevertheless, the likelihood of this possibility is somewhat attenuated as the findings largely aligned with predictions which arose from existing psychological and linguistic research as well as theory from evolutionary human science. To explain the findings as resulting from differences in canonicity, it would be necessary to posit how the sampling strategy used for homosexual male and female authors biassed language use in opposite directions in each sample in a manner which is consistent with the theoretical hypotheses and predictions. Although the non-heterosexual samples comprised novels that were published much more recently than the novels in the heterosexual samples, those differences in publication year were controlled for in all analyses. Correlations between publication year and all psycholinguistic outcome variables are available in the Supplementary Materials, as are correlations between authors’ age at publication and all the psycholinguistic outcome variables (Supplementary Tables 8, 10, 11).

The group differences reported in the study could be somewhat attenuated because of the diversity of author demographics included in the samples of novelists. For example, authors were sampled from more than five countries. Authors’ age in the heterosexual sample of 304 novels varied from 24 to 68, while year of publication varied from 1801 to 2017 (Luoto and van Cranenburgh, 2021). Likewise, although the sample comprised mainly Caucasian authors, the full sample included authors whose racial backgrounds were Latino, African–American, Asian, Native American, and mixed (see Supplementary Tables 2–6 for details). Though making the sample more representative of the respective authors’ populations, this sample diversity may have caused more variation in the psycholinguistic outcome variables than studying more homogenous author populations, and this higher variation could have resulted in smaller effect sizes (as in Newman et al., 2008). Thus, the effect sizes reported in this study could be underestimates, and having less variation in publication year, age, race, ethnicity, and nationality can lead to detecting larger effect sizes.

The authors’ sexual orientation was determined based on biographical information, including information on the sex of any partners (married or otherwise) that the authors had or any self-identification related to sexual orientation that the authors may have made publicly known (Luoto and van Cranenburgh, 2021). The authors’ sexual orientation for the purposes of this study is therefore based on both manifest sexual behaviour as well as self-identification; however, both sexual behaviour and sexual orientation may undergo various changes over time, especially in non-heterosexual women (Luoto et al., 2019a,b), which is why the use of an aggregate measure of lifetime sexual behaviour and sexual orientation may not accurately track a person’s sexual behaviour or sexual orientation at any single point in time. Sexual orientation is used in this study as an instructive overall indicator of an author’s sexual behaviour and attractions over their lifetimes, and as such may be limited by the availability of such information in biographical material (Luoto and van Cranenburgh, 2021).

One reason why the gender shift hypothesis was not strongly supported in homosexual female authors could have been because it was not possible to control for butch/femme differences in the sampled authors. This would have been an important addition to the study. After all, there can be significant variation in the masculinity/femininity of non-heterosexual women, and research on non-heterosexual women should take this variation, conceptualised, e.g., via butch/femme categories, into account by analysing different groups of non-heterosexual women separately (Luoto et al., 2019a,b). However, in this research on literary fiction, it would have been difficult (if not impossible) to study women’s self-identification as masculine butches or feminine femmes because many of the authors had passed away.

## Conclusion

These findings add to prior psychobehavioural and linguistic research in four main ways: (1) the results show the existence of psycholinguistic sex differences and, for the first time, psycholinguistic sexual orientation differences on a greater temporal continuum, in a way that reflects existing findings from cognitive and behavioural sciences in contemporary populations; (2) the findings are derived from culturally esteemed material which has hardly been touched by psychologists and evolutionary scientists working on sex differences and sexual orientation differences; (3) the findings build on a theoretical framework from evolutionary life sciences and cognitive neuroscience, which is seldom utilised by linguists or literary scholars; and (4) the findings are derived using a psycholinguistic methodological approach on literary Big Data which taps into the study of language and literary art as windows into the intricacies of human minds.

## Data Availability Statement

The datasets generated for this study can be found in online repositories (Luoto and van Cranenburgh, 2021). The names of the repository/repositories and accession number(s) can be found below: https://doi.org/10.1016/j.dib.2020.106655.

## Ethics Statement

Ethical review and approval was not required for the study on human participants in accordance with the local legislation and institutional requirements. Written informed consent for participation was not required for this study in accordance with the national legislation and the institutional requirements.

## Author Contributions

SL designed the study, acquired the funding, collected and analysed the data, prepared the data visualisations, and wrote the manuscript.

## Conflict of Interest

The author declares that the research was conducted in the absence of any commercial or financial relationships that could be construed as a potential conflict of interest.

## Footnotes

1 The only exceptions to this publication range were three novels by Aphra Behn, a bisexual female author whose three novels included in this study were published in 1688–1689. These novels were included to increase the sample size of novels written by bisexual female authors.

2 A note on a few novelists and novels that were preliminarily chosen from the literary anthologies and biographical guides but finally excluded from the study: Ernest Dowson was excluded because he wrote collaborative novels; Lewis Carroll was excluded because of a substantial divergence from realism in his novels; William Morris’s novels were excluded because they were published when he was significantly older than the age of 45 used in the selection criteria; although several novels by Jeanette Winterson were included, *The Battle of the Sun* and *Weight* were omitted because they are primarily children’s literature which may have slightly confounded the results because of a different writing style.

3 In some instances, an author was married to an opposite-sex partner but was still coded as homosexual. This was the case for example with Virginia Woolf, who reportedly felt no physical attraction for her husband, and many biographers agree that it was essentially a sexless marriage. Her most notable affair was with Vita Sackville-West.

4 URLs respectively: http://www.gutenberg.org/ http://gutenberg.net.au/ https://archive.org/

5 There were four other psycholinguistic categories (sexual, ≥6 letters, conjunctions, and negative emotions) in which sex differences were not statistically significant (see Figure 1 and Supplementary Table 8). However, the sex difference effect sizes (0.20 ≤ *d* ≤ 0.24) for these variables were non-trivial, which is why they are not indicated with black bars in Figures 2, 3. With this in mind, I emphasise the need for future research with adequately powered and focussed samples to further explore whether sex differences in those word categories that were non-trivial but statistically non-significant in the current study (i.e., sexual words, words with ≥6 letters, conjunctions, and negative emotions) can be reliably found in other larger samples.

6 I use the term ‘sexual dimorphism’ in the traditional evolutionary biological sense of referring to sexually differentiated traits which may nevertheless have overlapping distributions between the sexes.
