# Supplementary material for: Sexual Dimorphism in Language, and the Gender Shift Hypothesis of Homosexuality
Source: Front Psychol. 2021 May 31;12:639887. doi: 10.3389/fpsyg.2021.639887 (PMC8200855; doi:10.3389/fpsyg.2021.639887)
Supplement: Supplementary file 1 [file Data_Sheet_1.docx]

Supplementary Materials for

Sexual dimorphism in language, and the gender shift hypothesis of homosexuality

**This file includes:**

Hypotheses and Predictions

Supplementary Text

Tables S1 to S11

**Other Supplementary Materials for this manuscript include the following:**

Psycholinguistic data used in this study are available in Luoto and van Cranenburgh (2021).

**Hypotheses and Predictions**

The main hypothesis of this study was that psychological sex differences and sexual orientation differences, as reported in psychological research on people living in the 21st century (Archer, 2019; Luoto, Krams, & Rantala, 2019; Xu, Norton, & Rahman, 2017), replicate or have equivalents in the linguistic outputs of authors of literature living and writing decades and centuries ago. Since literary art is solely produced by the human mind—or minds—it reflects the general inclinations and idiosyncrasies with which that mind or those minds are preoccupied (Carroll, 2018; Muthukrishna, Doebeli, Chudek, & Henrich, 2018; Pennebaker & Ireland, 2011). It is therefore possible to gain invaluable insight into human nature, and additional evidence on psychological sex differences and sexual orientation differences, by analysing works of literature.

Fifteen predictions on sex differences and sexual orientation differences were made based on existing research in psychology, cognitive neuroscience, and linguistics (e.g. Archer, 2019; Luoto et al., 2019; Xu et al., 2017). The predictions are listed in Table S1. A preregistration of the hypotheses was made in August 2018 and is available at <http://aspredicted.org/blind.php?x=up3i96>. Specific predictions were not preregistered as they were subsequently derived from the hypotheses and existing literature.

Supplementary Text

Psycholinguistic categories

The following LIWC categories were used in the analyses. Examples of words in each category are given. The examples comprise words actually used in the novels in this sample.

1. **Analytical thinking**. This variable is a factor-analytically derived dimension based on eight function word dimensions. All eight function word categories load on a single dimension: two positively (articles, prepositions) and six negatively (personal pronouns, impersonal pronouns, auxiliary verbs, conjunctions, adverbs, and negations). A high value on this dimension reflects formal, logical, and hierarchical thinking; lower values reflect more informal, personal, here-and-now, and narrative thinking. See Pennebaker et al. (2014).
2. **Words with six or more letters** (e.g. ‘ponderous’)
3. **Personal pronouns** (e.g. ‘I’, ‘you’, ‘his’, ‘her’, ‘herself’, ‘ours’)
4. **Articles** (‘the’, ‘a’, ‘an’)
5. **Positive emotion** (e.g. ‘good’, ‘bliss’, ‘merry’, ‘smile’, ‘beloved’)
6. **Negative emotion** (e.g. ‘ruin’, ‘mourn’, ‘resent’, ‘depressed’, ‘crying’)
7. **Anxiety** (e.g. ‘fearful’, ‘stresses’, ‘worry’, ‘apprehension’)
8. **Anger** (e.g. ‘revenge’, ‘punish’, ‘furious’, ‘murder’, ‘outrage’)
9. **Sad** (e.g. ‘misery’, ‘depressed’, ‘cry’, ‘sorrow’)
10. **Social** (e.g. ‘trust’, ‘love’, ‘dear’, ‘darling’, ‘sociable’, ‘politely’, ‘compassion’)
11. **Cognitive processes** (e.g. ‘memory’, ‘idea’, ‘realize’, ‘know’, ‘hence’, ‘thoughts’)
12. **Differentiation** (e.g. ‘either’, ‘or’, ‘however’, ‘versus’, ‘unless’, ‘nevertheless’)
13. **Conjunctions** (e.g. ‘but’, ‘and’, ‘so’, ‘how’, ‘then’, ‘because’)
14. **Sexual** (e.g. ‘lusty’, ‘slut’, ‘lover’, ‘fuck’, ‘lust’, ‘orgasm’)
15. **Death** (e.g. ‘war’, ‘mortals’, ‘dying’, ‘dead’, ‘killing’, ‘murderous’)
16. **Verbs** (e.g. ‘go’, ‘be’, ‘listen’, ‘trying’)
17. **Past focus** (including past tense verbs and words related to the past, e.g. ‘previously’, ‘yesterday’, ‘was’, ‘had’, ‘done’)
18. **Present focus** (including present tense verbs and words related to the present, e.g. ‘today’, ‘attract’, ‘hate’, ‘thank’)
19. **Future focus** (e.g. ‘will’, ‘shall’, ‘expectation’, ‘foresee’, ‘prospect’, ‘anticipation’)
20. **Swear words** (e.g. ‘cunt’, ‘fuck’, ‘whore’)
21. **Numbers** (e.g. ‘1’, ‘twenty’, ‘279’, ‘thousands’)
22. **Risk** (e.g. ‘distrusting’, ‘apprehension’, ‘hazard’, ‘disaster’)
23. **Space** (e.g. ‘wherever’, ‘down’, ‘surrounding’, ‘land’, ‘middle’, ‘right’, ‘skyline’)
24. **Work** (e.g. ‘earns’, ‘company’, ‘colleague’, ‘profit’, ‘duty’, ‘financial’)^[[1]](#footnote-1)^

Additional statistical tests

Effect sizes (Cohen’s *d*s) for *t* tests were calculated (Table S9) using an online effect size calculator (Becker, 2000): these calculations were based on the means and standard deviations that were acquired using SPSS version 26.

Homogeneity of variances of residuals was analysed using Levene’s test. The data were further checked for normality of distribution of residuals using the Shapiro-Wilk test and skewness analyses in SPSS. Data that had non-normally distributed residuals (i.e. skewness > 1; or skewness > skewness Std. Error multiplied by 2; or *p*-value < .05 in the Shapiro-Wilk test) were natural logarithm transformed to reach normal distribution. If the logarithm transformed data did not meet the assumptions of normality and homogeneity of variances of residuals, Whitney-Mann *U* test was used to calculate the effect size *r* statistic using the formula *r* = *Z* / √*n*. For ease of comparison, the *r* value was transformed into *d* (Table S9) using an effect size calculator (Lenhard & Lenhard, 2016). Outlier analysis was conducted using Cook’s distance and boxplot graphs produced in SPSS version 26.

There are various ways to report the results of multilevel analysis depending on the parameters of the analysis. It is important to note the complexity of this topic (Hedges, 2007; Nakagawa & Cuthill, 2007), the various existing suggestions for reporting effect sizes in multilevel models (Baguley, 2009; Feingold, 2015), and the lack of full agreement on how to do it (Lai & Kwok, 2016). There is no consensus among statisticians on which effect size to use in multilevel modelling (Garson, 2014). Some authors have suggested that the unstandardised beta can be used to calculate *d* using pooled standard deviation and the following formula: *d* = *b* / *SD*_pooled_ (Baguley, 2009; Feingold, 2015). This protocol was followed in this study.

## Additional tests on cognitive process words and analytical thinking

This post-hoc analysis included social words, positive emotion words, anxiety words, and sadness-related words as covariates in a multilevel model on sex differences in cognitive process words. In this model, the effect of sex on cognitive process word frequency disappears: *γ* = –0.083, *SE* = 0.193, *t*(193) = –0.431, *p* = .667, 95%CI = [–0.46; 0.30]. Running the same multilevel model on analytical thinking as the outcome variable shows that positive emotion words (*t*(292) = –6.067, *p* < .001) and social words (*t*(296) = –6.564, *p* < .001) are significantly *negatively* correlated with analytical thinking. This is interesting because positive emotion words (*t*(293) = 10.055, *p* < .001) and anxiety-related words (*t*(293) = 3.186, *p* = .002) are *positively* correlated with cognitive process words in these data. The sex difference in analytical thinking becomes marginally non-significant, *γ* = 2.848, *SE* = 1.628, *t*(192) = 1.749, *p* = .082, 95%CI = [–0.36; 6.06], when social words, positive emotion words, anxiety words, and sadness-related words are added as covariates.

## Additional tests on time orientation and verb frequency

The results of the main analyses suggested that female authors’ seemingly higher focus on present and future was likely driven by their higher verb use. Therefore, further multilevel analyses were conducted in which verb frequency was added as a covariate when analysing sex differences in present focus and future focus. The effect of author’s sex on present focus (logarithm transformed) was no longer significant *γ* = –0.035, *SE* = 0.033, *t*(178) = –1.073, *p* = .285, 95%CI = [–0.10; 0.03], but verb frequency was: *γ* = 0.063, *SE* = 0.007, *t*(299) = 9.580, *p* < .001, 95%CI = [0.05; 0.08]. Likewise with future focus: the effect of author’s sex on future focus (logarithm transformed) was not significant *γ* = –0.024, *SE* = 0.029, *t*(174) = –0.836, *p* = .404, 95%CI = [–0.08; 0.03]. Verb frequency, however, was a significant predictor of future focus: *γ* = 0.036, *SE* = 0.005, *t*(296) = 6.816, *p* < .001, 95%CI = [0.03; 0.05]. These results indicate that female authors’ higher focus on present and future was indeed almost fully driven by female authors’ higher verb use. Past, present, and future focus were therefore summarised and each category was analysed as a percentage value of the total sum (past/present/future focus) by dividing it with the sum of the three categories. This analysis enables the evaluation of *relative* *within-sex* *focus* on past/present/future. For male authors, the distributions were as follows: 48.6% past, 44% present, and 7.4% future focus. For female authors, the distributions were 46.6% past, 45.9% present, and 7.5% future focus. These results show small sex differences, with male authors having a slightly higher relative focus on the past, while female authors had a slightly higher relative focus on the present. Relative future focus showed no substantial sex difference.

Table S1. Predictions based on a review of psychological, linguistic, and cognitive neuroscience research.

| **Sex difference predictions Existing evidence**  Prediction 1: *There is a small sex difference in analytical thinking which would show up in male authors’ word use as a relatively higher frequency of words in the LIWC category ‘Analytical thinking’, as well as using fewer conjunctions, fewer personal pronouns, more differentiation words, and more cognitive words than female authors.* | Men have higher levels of systemising than women; that is, men have a higher drive to analyse or construct systematic relationships in non-social domains (Baron-Cohen, Knickmeyer, & Belmonte, 2005; Groen, Fuermaier, Tucha, Koerts, & Tucha, 2018; Nettle, 2007; Zheng & Zheng, 2015). Perhaps surprisingly, Koolen (2018, p. 139) reported an effect size of *d* = –0.69, which indicated higher use of words reflecting cognitive processes in women than in men. Pennebaker et al. (2014) found that conjunctions and personal pronouns load negatively on the ‘analytical thinking’ factor (referred to as the ‘categorical-dynamic index’). Female bloggers have been reported to use significantly more conjunctions than males (Argamon, Konnel, Pennebaker, & Schier, 2007). |
| --- | --- |
| Prediction 2: *Men use words comprising six or more letters with higher frequency than women.* | Newman et al. (2008) reported a higher frequency of words with six or more letters in men’s language (*d* = 0.24). |
| Prediction 3: *Female authors’ word use focuses on people (personal pronouns and social words in LIWC) more than male authors’ language, while focus on objects (articles in LIWC) is more frequent in male authors’ language.* | This was one of the most robust differences reported by Newman et al. (2008) in English and Koolen (2018, p. 139) in Dutch. Using a sample of 14 000 texts, Newman et al. (2008) found that men used significantly more articles than women, but women used significantly more pronouns than men. Women also used more social words than men. This finding has been replicated using other languages (Argamon, Goulain, Horton, & Olsen, 2009; Koolen, 2018; Olsen, 2005) and other psychological methods (Lippa, 2010a, 2010b; Su, Rounds, & Armstrong, 2009). |
| Prediction 4: *Women’s word use has a relatively higher frequency of emotion words than men’s, with the exception of anger, which is predicted to be more frequent in men’s word use because of sex differences in aggression (Archer, 2009).* | Mehl and Pennebaker (2003) and Koolen (Koolen, 2018) found that women used more references to positive emotion, but men referred more to anger. Using a topic modelling approach, Jockers (2013, p. 136) reported that the topic of ‘Affection and happiness’ was more than twice as prevalent in female authors’ works than in those of male authors. Archer (2019) reported higher frequency of positive emotions (*d* = –0.20) and neuroticism (*d* = –0.31) in women, with no sex difference in negative emotions (*d* = 0.03). |
| Prediction 5: *Women’s word use has a relatively higher frequency of anxiety-related words than men’s word use.* | Women tend to have higher psychological anxiety scores than men (*d* = –0.59: Archer, 2019). Newman et al. (2008) found that women used more anxiety-related language than men (*d* = –0.16). |
| Prediction 6: *Men’s word use has a relatively higher frequency of words related to death than women’s word use.* | It was already Darwin (1859, 1871) who noted the greater proneness to physical aggression by men than women as part of a general mammalian pattern; see Archer (2009) for a comprehensive review on sex differences in aggression in humans. There is a significantly greater likelihood for males to suffer death from external causes at a young age (Archer, 2019; Gottschall, 2008), and sex differences in longevity (favouring females) are well-known across countries, time periods, and even species (Austad, 2006; Austad & Fischer, 2016). |
| Prediction 7: *Men’s word use has a relatively higher frequency of words related to sex than women’s.* | Men, on average, have faster life history strategies and higher sociosexuality than women: this basic life history difference has been found in numerous studies (Luoto et al., 2019). Newman and colleagues (2008), however, did not find significant differences between men and women in word frequencies related to sex. |
| Prediction 8: *There is a small sex difference in time orientation so that men’s word use has a relatively higher frequency of words related to the present (T1.1.2) while women focus slightly more on the future (T1.1.3)* | Men’s faster life history strategies could lead to men having higher present orientation relative to women. Empirical evidence suggests that women have higher cognitive inhibition capacity than men (Sjoberg & Cole, 2018). Newman et al. (2008), however, reported no significant sex differences in future tense verb use. Perhaps surprisingly, women were found to use *more* present tense verb forms than men (Newman et al., 2008). |
| Prediction 9: *Men use more swear words than women* | Newman et al. (2008) reported an effect size of 0.22 for swear words. The use of swear words is significantly predicted by circulating testosterone levels in men (Mascaro et al., 2018), suggesting a connection with masculinity and aggression. |
| Prediction 10: *Men use more numerical words than women* | Koolen (2018) reported greater male use of numbers in Dutch literature (*d* = 0.86). In academic publishing, Mathematics has a higher frequency of male than female authors (Thelwall, Bailey, Makita, Sud, & Madalli, 2019). See Archer (Archer, 2019) for a detailed discussion of sex differences in mathematical ability. |
| Prediction 11: *Men use more words related to occupation than women* | Koolen (2018) reported greater male use of occupational words in Dutch literature (*d* = 0.75). Newman et al. (2008) found a smaller effect size (*d* = 0.12) in a broader sample of spoken and written language. Parental investment theory suggests men can increase their fitness by partitioning resources to their offspring and partner, which means men, relative to women, may have higher fitness payoffs when they engage in occupational pursuits (Luoto, 2019a). |
| Prediction 12: *Men use more words related to risk than women* | Archer’s (2019) meta-analysis reported greater risk-taking in men (*d* = 0.49). |
| Prediction 13: *Men use more words related to space than women* | Men have better mental rotation skills (*d* = 0.66), visuospatial abilities (*d* = 0.48), and spatial visualisation (*d* = 0.23) than women (Archer, 2019). Although women have slightly better object location memory than men (*d* = –0.16), men have generally a better performance on memory tasks related to space, including spatial routes (*d* = 0.24) (Asperholm, Högman, Rafi, & Herlitz, 2019). Nevertheless, Newman et al. (2008) reported only a small sex difference in spatial words, favouring men (*d* = 0.12). |
| **Sexual orientation predictions Existing evidence** |  |
| Prediction 14: *Nonheterosexual female authors show psycholinguistic masculinisation in word categories in which sex differences have been previously recorded* | Nonheterosexual female authors are masculinised on a variety of psychological and biobehavioural traits (Luoto et al., 2019). These include a variety of measures used to assess verbal fluency: homosexual women have sex-atypical (masculinised) linguistic performance on three verbal fluency measures (Rahman, Abrahams, & Wilson, 2003). |
| Prediction 15: *Homosexual male authors show psycholinguistic feminisation in word categories in which sex differences have been previously recorded* | Homosexual men show highly feminised patterns of verbal fluency (Rahman et al., 2003) and have sex-atypical (feminised) cognitive performance (Xu et al., 2017). |

## Table S2. List of heterosexual male novelists

| Author | Novel | Publication year | Country | Race |
| --- | --- | --- | --- | --- |
| Walter Scott | *Waverley* | 1814 | SCT | Cau |
| Walter Scott | *The Black Dwarf* | 1816 | SCT | Cau |
| James Hogg | *The Brownie of Bodsbeck* | 1817 | SCT | Cau |
| John Galt | *Annals of the Parish* | 1821 | SCT | Cau |
| John Galt | *Ringan Gilhaize* | 1823 | SCT | Cau |
| Thomas Love Peacock | *Maid Marian* | 1822 | ENG | Cau |
| Thomas Love Peacock | *Crotchet Castle* | 1831 | ENG | Cau |
| James Fenimore Cooper | *The Pilot: A Tale of the Sea* | 1824 | US | Cau |
| James Fenimore Cooper | *The Headsman: The Abbaye des Vignerons* | 1833 | US | Cau |
| Thomas Moore | *The Epicurean* | 1827 | IRL | Cau |
| Edgar Allan Poe | *The Narrative of Arthur Gordon Pym of Nantucket* | 1838 | US | Cau |
| Charles Dickens | *Dombey and Son* | 1846 | ENG | Cau |
| Charles Dickens | *A Tale of Two Cities* | 1859 | ENG | Cau |
| John Henry Newman | *Loss and Gain* | 1848 | ENG | Cau |
| John Henry Newman | *Callista* | 1855 | ENG | Cau |
| Nathaniel Hawthorne | *The Scarlet Letter* | 1850 | US | Cau |
| Nathaniel Hawthorne | *The House of the Seven Gables* | 1851 | US | Cau |
| Herman Melville | *Moby-Dick; or, The Whale* | 1851 | US | Cau |
| Herman Melville | *The Confidence-Man: His Masquerade* | 1857 | US | Cau |
| William Wells Brown | *Clotel; or, The President's Daughter* | 1853 | US | Bla |
| Frank J. Webb | *The Garies and Their Friends* | 1857 | US | Bla |
| George Meredith | *Rhoda Fleming* | 1865 | ENG | Cau |
| George Meredith | *Beauchamp's Career* | 1875 | ENG | Cau |
| William Dean Howells | *Their Wedding Journey* | 1872 | US | Cau |
| William Dean Howells | *A Modern Instance* | 1882 | US | Cau |
| Thomas Hardy | *Far from the Madding Crowd* | 1874 | ENG | Cau |
| Thomas Hardy | *The Mayor of Casterbridge* | 1886 | ENG | Cau |
| Mark Twain | *The Adventures of Tom Sawyer* | 1876 | US | Cau |
| Mark Twain | *Adventures of Huckleberry Finn* | 1884 | US | Cau |
| George Washington Cable | *The Grandissimes* | 1880 | US | Cau |
| George Washington Cable | *Bonaventure: A Prose Pastoral of Acadian Louisiana* | 1888 | US | Cau |
| Robert Louis Stevenson | *Kidnapped* | 1886 | SCT | Cau |
| Robert Louis Stevenson | *Catriona* | 1893 | SCT | Cau |
| William Sharp | *Pharais* | 1894 | SCT | Cau |
| William Sharp | *Green Fire: A Romance* | 1896 | SCT | Cau |
| Stephen Crane | *The Red Badge of Courage* | 1895 | US | Cau |
| Stephen Crane | *Active Service* | 1899 | US | Cau |
| Rudyard Kipling | *Captains Courageous* | 1896 | ENG | Cau |
| Charles W. Chesnutt | *The House Behind the Cedars* | 1900 | US | Mix |
| Charles W. Chesnutt | *The Colonel's Dream* | 1905 | US | Mix |
| Theodore Dreiser | *Sister Carrie* | 1900 | US | Cau |
| Theodore Dreiser | *The "Genius"* | 1915 | US | Cau |
| Frank Norris | *The Octopus: A Story of California* | 1901 | US | Cau |
| Frank Norris | *The Pit: A Story of Chicago* | 1903 | US | Cau |
| Rudyard Kipling | *Kim* | 1901 | ENG | Cau |
| Upton Sinclair | *The Jungle* | 1906 | US | Cau |
| Upton Sinclair | *They Call Me Carpenter* | 1922 | US | Cau |
| Ford Madox Ford | *The Fifth Queen Crowned* | 1908 | ENG | Cau |
| Ford Madox Ford | *The Good Soldier* | 1915 | ENG | Cau |
| Jack London | *Martin Eden* | 1909 | US | Cau |
| Jack London | *Michael, Brother of Jerry* | 1917 | US | Cau |
| G. K. Chesterton | *The Ball and The Cross* | 1909 | ENG | Cau |
| G. K. Chesterton | *The Flying Inn* | 1914 | ENG | Cau |
| Walter de la Mare | *The Return* | 1910 | ENG | Cau |
| Walter de la Mare | *Memoirs of a Midget* | 1921 | ENG | Cau |
| W. E. B. Du Bois | *The Quest of the Silver Fleece* | 1911 | US | Bla |
| Sherwood Anderson | *Windy McPherson's Son* | 1916 | US | Cau |
| Sherwood Anderson | *Poor White* | 1920 | US | Cau |
| James Joyce | *A Portrait of the Artist as a Young Man* | 1916 | IRL | Cau |
| James Joyce | *Ulysses* | 1922 | IRL | Cau |
| Sinclair Lewis | *Main Street* | 1920 | US | Cau |
| Sinclair Lewis | *Dodsworth* | 1929 | US | Cau |
| Jean Toomer | *Cane* | 1923 | US | Bla |
| Ernest Hemingway | *A Farewell to Arms* | 1929 | US | Cau |
| Ernest Hemingway | *The Old Man and the Sea* | 1952 | US | Cau |
| Bernard Malamud | *The Natural* | 1952 | US | Cau |
| Bernard Malamud | *The Fixer* | 1966 | US | Cau |
| William Golding | *Lord of the Flies* | 1954 | ENG | Cau |
| William Golding | *Pincher Martin* | 1956 | ENG | Cau |
| Kingsley Amis | *Lucky Jim* | 1954 | ENG | Cau |
| Kingsley Amis | *Colonel Sun* | 1968 | ENG | Cau |
| Norman Mailer | *The Deer Park* | 1955 | US | Cau |
| Norman Mailer | *Why Are We in Vietnam?* | 1967 | US | Cau |
| Peter Matthiessen | *At Play in the Fields of the Lord* | 1965 | US | Cau |
| Paul Scott | *The Jewel in the Crown* | 1966 | ENG | Cau |
| P. H. Newby | *Something to Answer For* | 1968 | ENG | Cau |
| John Updike | *Couples* | 1968 | US | Cau |
| John Updike | *Marry Me* | 1976 | US | Cau |
| Philip Roth | *Portnoy's Complaint* | 1969 | US | Cau |
| Philip Roth | *The Professor of Desire* | 1977 | US | Cau |
| V. S. Naipaul | *In a Free State* | 1971 | TT-ENG | Asian |
| Don DeLillo | *Americana* | 1971 | US | Cau |
| Don DeLillo | *White Noise* | 1985 | US | Cau |
| John Berger | *G.* | 1972 | ENG | Cau |
| John Berger | *Pig Earth* | 1979 | ENG | Cau |
| David Storey | *A Temporary Life* | 1973 | ENG | Cau |
| David Storey | *Saville* | 1976 | ENG | Cau |
| Cormac McCarthy | *Child of God* | 1973 | US | Cau |
| Cormac McCarthy | *Blood Meridian* | 1985 | US | Cau |
| Stanley Middleton | *Holiday* | 1974 | ENG | Cau |
| John Banville | *Nightspawn* | 1971 | IRL | Cau |
| John Banville | *The Book of Evidence* | 1989 | IRL | Cau |
| Larry McMurtry | *Terms of Endearment* | 1975 | US | Cau |
| Larry McMurtry | *Lonesome Dove* | 1985 | US | Cau |
| Richard Ford | *A Piece of My Heart* | 1976 | US | Cau |
| Richard Ford | *Independence Day* | 1995 | US | Cau |
| William Kennedy | *Billy Phelan's Greatest Game* | 1978 | US | Cau |
| E. L. Doctorow | *Billy Bathgate* | 1989 | US | Cau |
| Julian Barnes | *Metroland* | 1980 | ENG | Cau |
| Julian Barnes | *Flaubert's Parrot* | 1984 | ENG | Cau |
| Salman Rushdie | *Midnight's Children* | 1981 | ENG-IND | Asian |
| Ian McEwan | *The Comfort of Strangers* | 1981 | ENG | Cau |
| Ian McEwan | *Black Dogs* | 1992 | ENG | Cau |
| Pete Dexter | *God's Pocket* | 1983 | US | Cau |
| Pete Dexter | *The Paperboy* | 1995 | US | Cau |
| Graham Swift | *Waterland* | 1983 | ENG | Cau |
| Graham Swift | *Last Orders* | 1996 | ENG | Cau |
| Denis Johnson | *Angels* | 1983 | US | Cau |
| Denis Johnson | *Resuscitation of a Hanged Man* | 1991 | US | Cau |
| Barry Unsworth | *Stone Virgin* | 1985 | ENG | Cau |
| Barry Unsworth | *Sacred Hunger* | 1992 | ENG | Cau |
| James Kelman | *A Chancer* | 1985 | SCT | Cau |
| James Kelman | *How Late It Was, How Late* | 1994 | SCT | Cau |
| Richard Russo | *Mohawk* | 1986 | US | Cau |
| Richard Russo | *The Risk Pool* | 1988 | US | Cau |
| Kazuo Ishiguro | *The Remains of the Day* | 1989 | ENG | Asian |
| Kazuo Ishiguro | *The Unconsoled* | 1995 | ENG | Asian |
| Edward St Aubyn | *Never Mind* | 1992 | ENG | Cau |
| Edward St Aubyn | *A Clue to the Exit* | 2000 | ENG | Cau |
| Jonathan Franzen | *Strong Motion* | 1992 | US | Cau |
| Jonathan Franzen | *The Corrections* | 2001 | US | Cau |
| Roddy Doyle | *Paddy Clarke Ha Ha Ha* | 1993 | IRL | Cau |
| Roddy Doyle | *A Star Called Henry* | 1999 | IRL | Cau |
| Jeffrey Eugenides | *The Virgin Suicides* | 1993 | US | Cau |
| Jeffrey Eugenides | *Middlesex* | 2002 | US | Cau |
| Will Self | *My Idea of Fun* | 1993 | ENG | Cau |
| Will Self | *The Butt* | 2008 | ENG | Cau |
| Jim Crace | *Signals of Distress* | 1994 | ENG | Cau |
| Jim Crace | *Being Dead* | 1999 | ENG | Cau |
| Richard Powers | *Galatea 2.2* | 1995 | US | Cau |
| Richard Powers | *The Time of Our Singing* | 2003 | US | Cau |
| Sebastian Barry | *The Whereabouts of Eneas McNulty* | 1998 | IRL | Cau |
| Sebastian Barry | *Annie Dunne* | 2002 | IRL | Cau |
| Colum McCann | *This Side of Brightness* | 1998 | IRL-US | Cau |
| Colum McCann | *Let the Great World Spin* | 2009 | IRL-US | Cau |
| Michael Chabon | *The Amazing Adventures of Kavalier & Clay* | 2000 | US | Cau |
| Michael Chabon | *The Final Solution* | 2004 | US | Cau |
| Colson Whitehead | *John Henry Days* | 2001 | US | Bla |
| Colson Whitehead | *Sag Harbor* | 2009 | US | Bla |
| Howard Jacobson | *The Making of Henry* | 2004 | ENG | Cau |
| Howard Jacobson | *Kalooki Nights* | 2006 | ENG | Cau |
| Anthony Doerr | *About Grace* | 2004 | US | Cau |
| Anthony Doerr | *All the Light We Cannot See* | 2014 | US | Cau |
| Junot Díaz | *The Brief Wondrous Life of Oscar Wao* | 2007 | DO-US | Lat |
| Adam Foulds | *The Quickening Maze* | 2009 | ENG | Cau |
| Stephen Kelman | *Pigeon English* | 2011 | ENG | Cau |
| Adam Johnson | *The Orphan Master's Son* | 2012 | US | Cau |
| Robert Olen Butler | *The Hot Country* | 2012 | US | Cau |
| Robert Olen Butler | *The Star of Istanbul* | 2013 | US | Cau |
| Phil Klay | *Redeployment* | 2014 | US | Cau |
| Sunjeev Sahota | *The Year of the Runaways* | 2015 | ENG | Asian |

## Table S3. List of heterosexual female novelists

| Author | Novel | Publication year | Country | Race |
| --- | --- | --- | --- | --- |
| Maria Edgeworth | *Belinda* | 1801 | ENG | Cau |
| Maria Edgeworth | *The Absentee* | 1812 | ENG/IRL | Cau |
| Jane Austen | *Sense and Sensibility* | 1811 | ENG | Cau |
| Jane Austen | *Persuasion* | 1817 | ENG | Cau |
| Susan Edmonstone Ferrier | *Marriage* | 1818 | SCT | Cau |
| Susan Edmonstone Ferrier | *The Inheritance* | 1824 | SCT | Cau |
| Mary Shelley | *The Fortunes of Perkin Warbeck* | 1830 | ENG | Cau |
| Mary Shelley | *Falkner* | 1837 | ENG | Cau |
| Emily Brontë | *Wuthering Heights* | 1847 | ENG | Cau |
| Anne Brontë | *Agnes Grey* | 1847 | ENG | Cau |
| Anne Brontë | *The Tenant of Wildfell Hall* | 1848 | ENG | Cau |
| Charlotte Brontë | *Jane Eyre* | 1847 | ENG | Cau |
| Charlotte Brontë | *Villette* | 1853 | ENG | Cau |
| Elizabeth Gaskell | *Mary Barton* | 1848 | ENG | Cau |
| Elizabeth Gaskell | *North and South* | 1855 | ENG | Cau |
| Harriet Beecher Stowe | *Uncle Tom's Cabin* | 1852 | US | Cau |
| Harriet Beecher Stowe | *Dred: A Tale of the Great Dismal Swamp* | 1856 | US | Cau |
| Harriet E. Wilson | *Our Nig* | 1859 | US | Bla |
| George Eliot | *Adam Bede* | 1859 | ENG | Cau |
| George Eliot | *Felix Holt, the Radical* | 1866 | ENG | Cau |
| Harriet Ann Jacobs | *Incidents in the Life of a Slave Girl* | 1861 | US | Bla |
| Rebecca Harding Davis | *Margret Howth* | 1861 | US | Cau |
| Rebecca Harding Davis | *John Andross* | 1874 | US | Cau |
| Elizabeth Drew Stoddard | *The Morgesons* | 1862 | US | Cau |
| Elizabeth Drew Stoddard | *Two Men* | 1865 | US | Cau |
| Frances Harper | *Minnie's Sacrifice* | 1869 | US | Bla |
| Frances Harper | *Sowing and Reaping* | 1876 | US | Bla |
| Louisa May Alcott | *An Old Fashioned Girl* | 1870 | US | Cau |
| Louisa May Alcott | *Rose in Bloom: A Sequel to Eight Cousins* | 1876 | US | Cau |
| María Ruiz de Burton | *Who Would Have Thought It?* | 1872 | US | Lat |
| María Ruiz de Burton | *The Squatter and the Don* | 1885 | US | Lat |
| Ouida | *Two Little Wooden Shoes* | 1874 | ENG | Cau |
| Ouida | *Othmar* | 1885 | ENG | Cau |
| Mrs. Humphry Ward | *Robert Elsmere* | 1888 | ENG | Cau |
| Mrs. Humphry Ward | *Sir George Tressady* | 1896 | ENG | Cau |
| Sarah Grand | *Ideala* | 1888 | IRL | Cau |
| Sarah Grand | *The Beth Book* | 1897 | IRL | Cau |
| Kate Chopin | *At Fault* | 1890 | US | Cau |
| Kate Chopin | *The Awakening* | 1899 | US | Cau |
| Emma Dunham Kelley | *Megda* | 1891 | US | Cau |
| May Sinclair | *Mr and Mrs Nevill Tyson* | 1898 | ENG | Cau |
| May Sinclair | *The Immortal Moment* | 1908 | ENG | Cau |
| Pauline Hopkins | *Hagar's Daughter: A Story of Southern Caste Prejudice* | 1901 | US | Bla |
| Pauline Hopkins | *Winona: A Tale of Negro Life in the South and Southwest* | 1902 | US | Bla |
| Edith Wharton | *The Valley of Decision* | 1902 | US | Cau |
| Edith Wharton | *The Fruit of the Tree* | 1907 | US | Cau |
| Katharine Tynan | *The Story of Bawn* | 1906 | IRL | Cau |
| Katharine Tynan | *Mary Gray* | 1909 | IRL | Cau |
| Ellen Glasgow | *The Ancient Law* | 1908 | US | Cau |
| Ellen Glasgow | *The Builders* | 1919 | US | Cau |
| Charlotte Perkins Gilman | *What Diantha Did* | 1909 | US | Cau |
| Charlotte Perkins Gilman | *The Crux* | 1911 | US | Cau |
| Susan Glaspell | *The Visioning* | 1911 | US | Cau |
| Susan Glaspell | *Fidelity* | 1915 | US | Cau |
| Dorothy Richardson | *Pointed Roofs* | 1915 | ENG | Cau |
| Dorothy Richardson | *The Tunnel* | 1919 | ENG | Cau |
| Rebecca West | *The Judge* | 1922 | ENG | Cau |
| Rebecca West | *The Thinking Reed* | 1936 | ENG | Cau |
| Jessie Redmon Fauset | *Plum Bun: A Novel Without a Moral* | 1928 | US | Bla |
| Nella Larsen | *Quicksand* | 1928 | US | Bla |
| Nella Larsen | *Passing* | 1929 | US | Bla |
| Zora Neale Hurston | *Their Eyes Were Watching God* | 1937 | US | Bla |
| Janet Lewis | *The Wife of Martin Guerre* | 1941 | US | Cau |
| Janet Lewis | *The Trial of Sören Qvist* | 1947 | US | Cau |
| Josefina Niggli | *Mexican Village* | 1945 | US | Lat |
| Josefina Niggli | *Step Down, Elder Brother* | 1947 | US | Lat |
| Nina Bawden | *Change Here for Babylon* | 1955 | ENG | Cau |
| Nina Bawden | *Just Like A Lady* | 1960 | ENG | Cau |
| Alice Childress | *Like One of the Family* | 1956 | US | Bla |
| Alice Childress | *A Hero Ain't Nothin' But A Sandwich* | 1973 | US | Bla |
| Muriel Spark | *Robinson* | 1958 | SCT | Cau |
| Muriel Spark | *The Prime of Miss Jean Brodie* | 1961 | SCT | Cau |
| Ruth Prawer Jhabvala | *The Householder* | 1960 | ENG-US | Cau |
| Doris Lessing | *The Golden Notebook* | 1962 | ENG-ZI | Cau |
| Doris Lessing | *The Summer Before the Dark* | 1973 | ENG-ZI | Cau |
| Bernice Rubens | *Madame Sousatzka* | 1962 | WAL | Cau |
| Lore Segal | *Other People's Houses* | 1964 | AU–US | Cau |
| Lore Segal | *Her First American* | 1985 | AU–US | Cau |
| Fay Weldon | *The Fat Woman's Joke* | 1967 | ENG | Cau |
| Fay Weldon | *Female Friends* | 1974 | ENG | Cau |
| Beryl Bainbridge | *Another Part of the Wood* | 1968 | ENG | Cau |
| Toni Morrison | *The Bluest Eye* | 1970 | US | Bla |
| Toni Morrison | *Sula* | 1973 | US | Bla |
| Anne Tyler | *Searching for Caleb* | 1975 | US | Cau |
| Anne Tyler | *Breathing Lessons* | 1988 | US | Cau |
| Carol Shields | *Small Ceremonies* | 1976 | US-CAN | Cau |
| Carol Shields | *The Box Garden* | 1977 | US-CAN | Cau |
| Rose Tremain | *Sadler's Birthday* | 1976 | ENG | Cau |
| Rose Tremain | *Restoration* | 1989 | ENG | Cau |
| Barbara Pym | *Quartet in Autumn* | 1977 | ENG | Cau |
| Penelope Lively | *The Road to Lichfield* | 1977 | ENG | Cau |
| Penelope Lively | *According to Mark* | 1984 | ENG | Cau |
| Penelope Fitzgerald | *The Bookshop* | 1978 | ENG | Cau |
| Penelope Fitzgerald | *Offshore* | 1979 | ENG | Cau |
| Marilynne Robinson | *Housekeeping* | 1980 | US | Cau |
| Marilynne Robinson | *Gilead* | 2004 | US | Cau |
| Alison Lurie | *Foreign Affairs* | 1984 | US | Cau |
| Hilary Mantel | *Every Day is Mother's Day* | 1985 | ENG | Cau |
| Hilary Mantel | *An Experiment in Love* | 1995 | ENG | Cau |
| Alice McDermott | *That Night* | 1987 | US | Cau |
| Alice McDermott | *Charming Billy* | 1998 | US | Cau |
| Deborah Levy | *Beautiful Mutants* | 1989 | ENG | Cau |
| Deborah Levy | *Billy and Girl* | 1999 | ENG | Cau |
| A. S. Byatt | *Possession* | 1990 | ENG | Cau |
| Pat Barker | *Regeneration* | 1991 | ENG | Cau |
| Pat Barker | *The Eye in the Door* | 1993 | ENG | Cau |
| Andrea Barrett | *The Middle Kingdom* | 1991 | US | Cau |
| Andrea Barrett | *The Forms of Water* | 1993 | US | Cau |
| Annie Proulx | *Postcards* | 1992 | US | Cau |
| Annie Proulx | *The Shipping News* | 1993 | US | Cau |
| Barbara Kingsolver | *Pigs in Heaven* | 1993 | US | Cau |
| Barbara Kingsolver | *The Poisonwood Bible* | 1998 | US | Cau |
| Shirley Ann Grau | *Roadwalkers* | 1994 | US | Cau |
| Anne Enright | *The Wig My Father Wore* | 1995 | IRL | Cau |
| Anne Enright | *The Gathering* | 2007 | IRL | Cau |
| Joyce Carol Oates | *We Were the Mulvaneys* | 1996 | US | Cau |
| Joyce Carol Oates | *Blonde* | 2000 | US | Cau |
| Nicola Barker | *Wide Open* | 1998 | ENG | Cau |
| Elizabeth Strout | *Amy and Isabelle* | 1998 | US | Cau |
| Jane Smiley | *Horse Heaven* | 2000 | US | Cau |
| Jane Smiley | *Good Faith* | 2003 | US | Cau |
| Geraldine Brooks | *Year of Wonders* | 2001 | AUS-US | Cau |
| Geraldine Brooks | *March* | 2005 | AUS-US | Cau |
| Marina Warner | *The Leto Bundle* | 2001 | ENG | Cau |
| Rachel Seiffert | *The Dark Room* | 2001 | ENG | Cau |
| Rachel Seiffert | *Afterwards* | 2007 | ENG | Cau |
| Julia Glass | *Three Junes* | 2002 | US | Cau |
| Julia Glass | *I See You Everywhere* | 2008 | US | Cau |
| Donna Tartt | *The Little Friend* | 2002 | US | Cau |
| Monica Ali | *Brick Lane* | 2003 | ENG | Mix |
| Monica Ali | *Untold Story* | 2011 | ENG | Mix |
| Jhumpa Lahiri | *The Namesake* | 2003 | US | Asian |
| Jhumpa Lahiri | *The Lowland* | 2013 | US | Asian |
| Lydia Millet | *Everyone's Pretty* | 2005 | US-CAN | Cau |
| Lydia Millet | *Pills and Starships* | 2014 | US-CAN | Cau |
| Zadie Smith | *On Beauty* | 2005 | ENG | Mix |
| Zadie Smith | *Swing Time* | 2016 | ENG | Mix |
| M. J. Hyland | *Carry Me Down* | 2006 | ENG | Cau |
| M. J. Hyland | *This is How* | 2009 | ENG | Cau |
| Nicola Barker | *Darkmans* | 2007 | ENG | Cau |
| Louise Erdrich | *The Plague of Doves* | 2008 | US | Nat |
| Louise Erdrich | *The Round House* | 2012 | US | Nat |
| Sarah Hall | *How to Paint a Dead Man* | 2009 | ENG | Cau |
| Sarah Hall | *The Wolf Border* | 2015 | ENG | Cau |
| Carol Birch | *Jamrach's Menagerie* | 2011 | ENG | Cau |
| Carol Birch | *Orphans of the Carnival* | 2016 | ENG | Cau |
| Jesmyn Ward | *Salvage the Bones* | 2011 | US | Bla |
| Jesmyn Ward | *Sing, Unburied, Sing* | 2017 | US | Bla |
| Alison Moore | *The Lighthouse* | 2012 | ENG | Cau |
| Alison Moore | *He Wants* | 2014 | ENG | Cau |
| Michèle Roberts | *Ignorance* | 2012 | ENG-FRA | Cau |
| Michèle Roberts | *The Walworth Beauty* | 2017 | ENG-FRA | Cau |
| C. E. Morgan | *The Sport of Kings* | 2016 | US | Cau |

## Table S4. List of homosexual male novelists

| Author | Novel | Publication year | Country | Race |
| --- | --- | --- | --- | --- |
| Samuel Butler | *Erewhon, or Over the Range* | 1872 | ENG | Cau |
| Samuel Butler | *The Way of All Flesh* | 1884 | ENG | Cau |
| Samuel Butler | *Erewhon Revisited* | 1901 | ENG | Cau |
| Henry Blake Fuller | *The Cliff-Dwellers* | 1893 | US | Cau |
| Henry Blake Fuller | *With the Procession* | 1895 | US | Cau |
| Henry Blake Fuller | *Under the Skylights* | 1901 | US | Cau |
| Charles Warren Stoddard | *For the Pleasure of His Company* | 1903 | US | Cau |
| E. F. Benson | *Scarlet and Hyssop* | 1902 | ENG | Cau |
| E. F. Benson | *An Act in a Backwater* | 1903 | ENG | Cau |
| E. F. Benson | *The Relentless City* | 1903 | ENG | Cau |
| E. F. Benson | *The Challoners* | 1904 | ENG | Cau |
| E. F. Benson | *The Angel of Pain* | 1905 | ENG | Cau |
| E. F. Benson | *The House of Defence* | 1906 | ENG | Cau |
| E. F. Benson | *The Blotting Book* | 1908 | ENG | Cau |
| E. F. Benson | *Daisy's Aunt* | 1910 | ENG | Cau |
| E. M. Forster | *Where Angels Fear to Tread* | 1905 | ENG | Cau |
| E. M. Forster | *The Longest Journey* | 1907 | ENG | Cau |
| E. M. Forster | *A Room with a View* | 1908 | ENG | Cau |
| E. M. Forster | *Howards End* | 1910 | ENG | Cau |
| E. M. Forster | *A Passage to India* | 1924 | ENG | Cau |
| Hugh Walpole | *The Wooden Horse* | 1909 | ENG | Cau |
| Hugh Walpole | *The Gods and Mr Perrin* | 1911 | ENG | Cau |
| Hugh Walpole | *The Prelude to Adventure* | 1912 | ENG | Cau |
| Hugh Walpole | *Fortitude* | 1913 | ENG | Cau |
| Hugh Walpole | *The Dark Forest* | 1916 | ENG | Cau |
| Hugh Walpole | *The Secret City* | 1919 | ENG | Cau |
| Hugh Walpole | *Jeremy* | 1919 | ENG | Cau |
| Hugh Walpole | *The Captives* | 1920 | ENG | Cau |
| Hugh Walpole | *The Cathedral* | 1922 | ENG | Cau |
| Ronald Firbank | *Vainglory* | 1915 | ENG | Cau |
| Ronald Firbank | *Inclinations* | 1916 | ENG | Cau |
| Ronald Firbank | *Caprice* | 1917 | ENG | Cau |
| C. H. B. Kitchin | *Crime at Christmas* | 1934 | ENG | Cau |
| Glenway Wescott | *The Pilgrim Hawk* | 1940 | US | Cau |
| Glenway Wescott | *Apartment in Athens* | 1945 | US | Cau |
| Denton Welch | *Maiden Voyage* | 1943 | ENG | Cau |
| Denton Welch | *In Youth is Pleasure* | 1945 | ENG | Cau |
| Denton Welch | *A Voice Through a Cloud* | 1950 | ENG | Cau |
| John Horne Burns | *The Gallery* | 1947 | US | Cau |
| Truman Capote | *Other Voices, Other Rooms* | 1948 | US | Cau |
| Truman Capote | *Summer Crossing* | 1950 | US | Cau |
| Truman Capote | *Breakfast at Tiffany's* | 1958 | US | Cau |
| Christopher Isherwood | *A Single Man* | 1964 | ENG | Cau |
| Angus Wilson | *Hemlock and After* | 1952 | ENG | Cau |
| Angus Wilson | *The Old Men at the Zoo* | 1961 | ENG | Cau |
| John Rechy | *City of Night* | 1963 | US | Lat |
| James Leo Herlihy | *Midnight Cowboy* | 1965 | US | Cau |
| James Baldwin | *Go Tell It on the Mountain* | 1953 | US | Bla |
| James Baldwin | *Giovanni's Room* | 1956 | US | Bla |
| James Baldwin | *Another Country* | 1962 | US | Bla |
| James Baldwin | *Tell Me How Long the Train's Been Gone* | 1968 | US | Bla |
| James Baldwin | *If Beale Street Could Talk* | 1974 | US | Bla |
| Patrick White | *The Living and the Dead* | 1941 | AUS | Cau |
| Patrick White | *The Tree of Man* | 1955 | AUS | Cau |
| Patrick White | *Voss* | 1957 | AUS | Cau |
| Patrick White | *The Solid Mandala* | 1966 | AUS | Cau |
| Patrick White | *The Vivisector* | 1970 | AUS | Cau |
| Patrick White | *The Eye of the Storm* | 1973 | AUS | Cau |
| Patrick White | *A Fringe of Leaves* | 1976 | AUS | Cau |
| Patrick White | *The Twyborn Affair* | 1979 | AUS | Cau |
| Sumner Locke Elliott | *Careful, He Might Hear You* | 1963 | AUS | Cau |
| Sumner Locke Elliott | *Fairyland* | 1990 | AUS | Cau |
| Desmond Hogan | *The Ikon Maker* | 1976 | IRL | Cau |
| Desmond Hogan | *The Leaves on Grey* | 1980 | IRL | Cau |
| Felice Picano | *The Lure* | 1979 | US | Cau |
| Felice Picano | *Late in the Season* | 1981 | US | Cau |
| Francis King | *Act of Darkness* | 1983 | ENG | Cau |
| George Whitmore | *Nebraska* | 1987 | US | Cau |
| Edmund White | *A Boy's Own Story* | 1982 | US | Cau |
| Edmund White | *Our Young Man* | 2016 | US | Cau |
| Christopher Bram | *Surprising Myself* | 1987 | US | Cau |
| Christopher Bram | *Hold Tight* | 1988 | US | Cau |
| Christopher Bram | *In Memory of Angel Clare* | 1989 | US | Cau |
| Armistead Maupin | *Tales of the City* | 1978 | US | Cau |
| Armistead Maupin | *More Tales of the City* | 1980 | US | Cau |
| Armistead Maupin | *Further Tales of the City* | 1982 | US | Cau |
| Armistead Maupin | *Babycakes* | 1984 | US | Cau |
| Armistead Maupin | *Significant Others* | 1987 | US | Cau |
| Armistead Maupin | *Sure of You* | 1989 | US | Cau |
| Armistead Maupin | *Maybe the Moon* | 1992 | US | Cau |
| Armistead Maupin | *The Night Listener* | 2000 | US | Cau |
| Armistead Maupin | *Michael Tolliver Lives* | 2007 | US | Cau |
| Armistead Maupin | *Mary Ann in Autumn* | 2010 | US | Cau |
| Armistead Maupin | *The Days of Anna Madrigal* | 2014 | US | Cau |
| Mark Richard Zubro | *Why Isn't Becky Twitchell Dead?* | 1991 | US | Cau |
| Mark Richard Zubro | *The Only Good Priest* | 1991 | US | Cau |
| Mark Richard Zubro | *The Principal Cause of Death* | 1992 | US | Cau |
| Mark Richard Zubro | *Political Poison* | 1994 | US | Cau |
| Mark Richard Zubro | *An Echo of Death* | 1995 | US | Cau |
| Mark Richard Zubro | *Rust On the Razor* | 1996 | US | Cau |
| Paul Lisicky | *Lawnboy* | 1998 | US | Cau |
| E. Lynn Harris | *If This World Were Mine* | 1998 | US | Bla |
| E. Lynn Harris | *Abide With Me* | 1999 | US | Bla |
| Steven Saylor | *Roman Blood* | 1991 | US | Cau |
| Steven Saylor | *Arms of Nemesis* | 1992 | US | Cau |
| Steven Saylor | *The Venus Throw* | 1995 | US | Cau |
| Steven Saylor | *A Mist of Prophecies* | 2002 | US | Cau |
| Patrick Gale | *The Aerodynamics of Pork* | 1985 | ENG | Cau |
| Patrick Gale | *Ease* | 1985 | ENG | Cau |
| Patrick Gale | *Kansas in August* | 1987 | ENG | Cau |
| Patrick Gale | *Facing the Tank* | 1988 | ENG | Cau |
| Patrick Gale | *The Facts of Life* | 1996 | ENG | Cau |
| Patrick Gale | *Tree Surgery for Beginners* | 1999 | ENG | Cau |
| Patrick Gale | *Rough Music* | 2000 | ENG | Cau |
| Patrick Gale | *A Sweet Obscurity* | 2003 | ENG | Cau |
| Patrick Gale | *Friendly Fire* | 2005 | ENG | Cau |
| David Leavitt | *The Lost Language of Cranes* | 1986 | US | Cau |
| David Leavitt | *Equal Affections* | 1989 | US | Cau |
| David Leavitt | *The Body of Jonah Boyd* | 2004 | US | Cau |
| David Leavitt | *The Indian Clerk* | 2007 | US | Cau |
| David Leavitt | *The Two Hotel Francforts* | 2013 | US | Cau |
| Jaime Manrique | *Our Lives Are the Rivers* | 2006 | COL-US | Lat |
| Jaime Manrique | *Cervantes Street* | 2012 | COL-US | Lat |
| Thomas Mallon | *Watergate* | 2012 | US | Cau |
| Thomas Mallon | *Finale* | 2015 | US | Cau |
| Michael Cunningham | *A Home at the End of the World* | 1990 | US | Cau |
| Michael Cunningham | *The Hours* | 1998 | US | Cau |
| Michael Cunningham | *Specimen Days* | 2005 | US | Cau |
| Michael Cunningham | *By Nightfall* | 2010 | US | Cau |
| Michael Cunningham | *The Snow Queen* | 2014 | US | Cau |
| Alan Hollinghurst | *The Swimming Pool Library* | 1988 | ENG | Cau |
| Alan Hollinghurst | *The Folding Star* | 1994 | ENG | Cau |
| Alan Hollinghurst | *The Spell* | 1998 | ENG | Cau |
| Alan Hollinghurst | *The Line of Beauty* | 2004 | ENG | Cau |
| Alan Hollinghurst | *The Stranger's Child* | 2011 | ENG | Cau |
| Alan Hollinghurst | *The Sparsholt Affair* | 2017 | ENG | Cau |
| Adam Mars-Jones | *Pilcrow* | 2008 | ENG | Cau |
| Gregory Maguire | *Lost* | 2001 | US | Cau |
| Gregory Maguire | *Mirror, Mirror* | 2003 | US | Cau |
| Gregory Maguire | *A Lion Among Men* | 2008 | US | Cau |
| Colm Tóibín | *The South* | 1990 | IRL | Cau |
| Colm Tóibín | *Brooklyn* | 2009 | IRL | Cau |
| Colm Tóibín | *Nora Webster* | 2014 | IRL | Cau |
| Paul Russell | *Boys of Life* | 1991 | US | Cau |
| Paul Russell | *The Unreal Life of Sergey Nabokov* | 2012 | US | Cau |
| Paul Russell | *Immaculate Blue* | 2015 | US | Cau |
| Chuck Palahniuk | *Fight Club* | 1996 | ENG | Cau |
| Chuck Palahniuk | *Invisible Monsters* | 1999 | ENG | Cau |
| Chuck Palahniuk | *Choke* | 2001 | ENG | Cau |
| Chuck Palahniuk | *Damned* | 2011 | ENG | Cau |
| Chuck Palahniuk | *Beautiful You* | 2014 | ENG | Cau |
| Chuck Palahniuk | *Make Something Up* | 2015 | ENG | Cau |
| Philip Hensher | *The Mulberry Empire* | 2002 | ENG | Cau |
| Philip Hensher | *The Northern Clemency* | 2008 | ENG | Cau |
| Philip Hensher | *King of the Badgers* | 2011 | ENG | Cau |
| Mark Gatiss | *The Vesuvius Club* | 2004 | ENG | Cau |
| Patrick Ness | *The Crash of Hennington* | 2003 | ENG-US | Cau |
| Patrick Ness | *The Crane Wife* | 2013 | ENG-US | Cau |
| Michael Sledge | *The More I Owe You* | 2010 | US | Cau |
| Andrew Sean Greer | *The Story of a Marriage* | 2008 | US | Cau |
| Andrew Sean Greer | *The Impossible Lives of Greta Wells* | 2013 | US | Cau |
| Dale Peck | *Martin and John* | 1993 | US | Cau |
| Dale Peck | *The Law of Enclosures* | 1996 | US | Cau |
| Dale Peck | *Now It's Time to Say Goodbye* | 1999 | US | Cau |
| Dale Peck | *The Garden of Lost and Found* | 2012 | US | Cau |
| Dale Peck | *Night Soil* | 2018 | US | Cau |
| Jonathan Kemp | *Ghosting* | 2015 | ENG | Cau |
| Adam Haslett | *Imagine Me Gone* | 2016 | US | Cau |
| Joe Okonkwo | *Jazz Moon* | 2016 | US | Bla |
| Bill Konigsberg | *Openly Straight* | 2013 | US | Cau |
| Christopher Rice | *The Flame* | 2014 | US | Cau |
| Christopher Rice | *Kiss The Flame* | 2015 | US | Cau |
| Christopher Rice | *Dance of Desire* | 2016 | US | Cau |
| Bill Konigsberg | *Honestly Ben* | 2016 | US | Cau |
| Brent Hartinger | *Three Truths and a Lie* | 2016 | US | Cau |
| Darryl Pinckney | *Black Deutschland* | 2016 | US | Bla |
| Joseph Olshan | *Black Diamond Fall* | 2018 | US | Cau |

## Table S5. List of homosexual female novelists

| Author | Novel | Publication year | Country | Race |
| --- | --- | --- | --- | --- |
| Amy Levy | *The Romance of a Shop* | 1888 | ENG | Cau |
| Selma Lagerlöf | *The Story of Gösta Berling* | 1891 | SWE | Cau |
| Selma Lagerlöf | *Jerusalem* | 1901 | SWE | Cau |
| Florence Converse | *Long Will* | 1903 | US | Cau |
| Florence Converse | *The Story of Wellesley* | 1915 | US | Cau |
| Renée Vivien | *A Woman Appeared to me* | 1904 | ENG | Cau |
| Gertrude Stein | *Three Lives* | 1909 | US | Cau |
| Gertrude Stein | *The Making of Americans* | 1925 | US | Cau |
| Gertrude Stein | *Ida: A Novel* | 1941 | US | Cau |
| Virginia Woolf | *The Voyage Out* | 1915 | ENG | Cau |
| Virginia Woolf | *Night and Day* | 1919 | ENG | Cau |
| Virginia Woolf | *Jacob's Room* | 1922 | ENG | Cau |
| Virginia Woolf | *Mrs Dalloway* | 1925 | ENG | Cau |
| Virginia Woolf | *To the Lighthouse* | 1927 | ENG | Cau |
| Virginia Woolf | *Orlando: A Biography* | 1928 | ENG | Cau |
| Virginia Woolf | *The Waves* | 1931 | ENG | Cau |
| Virginia Woolf | *Between the Acts* | 1941 | ENG | Cau |
| Radclyffe Hall | *The Unlit Lamp* | 1924 | ENG | Cau |
| Radclyffe Hall | *The Well of Loneliness* | 1928 | ENG | Cau |
| Sylvia Townsend Warner | *Lolly Willowes* | 1926 | ENG | Cau |
| Sylvia Townsend Warner | *Summer Will Show* | 1936 | ENG | Cau |
| Helen Rose Hull | *Islanders* | 1927 | US | Cau |
| Gale Wilhelm | *Torchlight to Valhalla* | 1938 | US | Cau |
| May Sarton | *The Bridge of Years* | 1946 | US | Cau |
| May Sarton | *A Shower of Summer Days* | 1952 | US | Cau |
| May Sarton | *The Small Room* | 1961 | US | Cau |
| May Sarton | *Kinds of Love* | 1970 | US | Cau |
| May Sarton | *As We Are Now* | 1973 | US | Cau |
| May Sarton | *Crucial Conversations* | 1975 | US | Cau |
| May Sarton | *A Reckoning* | 1978 | US | Cau |
| Patricia Highsmith | *Strangers on a Train* | 1950 | US | Cau |
| Patricia Highsmith | *The Price of Salt* | 1952 | US | Cau |
| Patricia Highsmith | *The Talented Mr. Ripley* | 1955 | US | Cau |
| Patricia Highsmith | *Ripley Under Ground* | 1970 | US | Cau |
| Patricia Highsmith | *Ripley's Game* | 1974 | US | Cau |
| Vin Packer | *Spring Fire* | 1952 | US | Cau |
| Vin Packer | *Come Destroy Me* | 1954 | US | Cau |
| Vin Packer | *Dark Don't Catch Me* | 1956 | US | Cau |
| Vin Packer | *The Evil Friendship* | 1958 | US | Cau |
| Vin Packer | *5:45 to Surburbia* | 1958 | US | Cau |
| Vin Packer | *The Girl on the Best Seller List* | 1960 | US | Cau |
| Rosemary Manning | *The Chinese Garden* | 1962 | ENG | Cau |
| Jane Rule | *Desert of the Heart* | 1964 | CAN | Cau |
| Jane Rule | *This Is Not for You* | 1970 | CAN | Cau |
| Jane Rule | *Against the Season* | 1971 | CAN | Cau |
| Jane Rule | *Contract With the World* | 1980 | CAN | Cau |
| Jane Rule | *Inland Passage* | 1985 | CAN | Cau |
| Jane Rule | *Memory Board* | 1987 | CAN | Cau |
| Maureen Duffy | *The Microcosm* | 1966 | ENG | Cau |
| Rita Mae Brown | *Rubyfruit Jungle* | 1973 | US | Cau |
| Rita Mae Brown | *In Her Day* | 1976 | US | Cau |
| Sandra Scoppettone | *Happy Endings Are All Alike* | 1978 | US | Cau |
| Audre Lorde | *Zami: A New Spelling of My Name* | 1982 | US | Bla |
| Katherine V. Forrest | *Curious Wine* | 1983 | US | Cau |
| Katherine V. Forrest | *Amateur City* | 1984 | US | Cau |
| Katherine V. Forrest | *Murder At The Nightwood Bar* | 1987 | US | Cau |
| Katherine V. Forrest | *The Beverly Malibu* | 1989 | US | Cau |
| Katherine V. Forrest | *Murder By Tradition* | 1991 | US | Cau |
| Katherine V. Forrest | *Liberty Square* | 1996 | US | Cau |
| Katherine V. Forrest | *Apparition Alley* | 1997 | US | Cau |
| Katherine V. Forrest | *Sleeping Bones* | 1999 | US | Cau |
| Katherine V. Forrest | *Hancock Park* | 2004 | US | Cau |
| Katherine V. Forrest | *High Desert* | 2013 | US | Cau |
| Valerie Miner | *Murder in the English Department* | 1982 | US | Cau |
| Jeanette Winterson | *Oranges Are Not the Only Fruit* | 1985 | ENG | Cau |
| Jeanette Winterson | *The Passion* | 1987 | ENG | Cau |
| Jeanette Winterson | *Sexing the Cherry* | 1989 | ENG | Cau |
| Jeanette Winterson | *Written on the Body* | 1992 | ENG | Cau |
| Jeanette Winterson | *Lighthousekeeping* | 2004 | ENG | Cau |
| Jeanette Winterson | *The Stone Gods* | 2007 | ENG | Cau |
| Jeanette Winterson | *The Daylight Gate* | 2012 | ENG | Cau |
| Ellen Galford | *The Fires of Bride* | 1986 | US/SCT | Cau |
| Valerie Miner | *All Good Women* | 1987 | US | Cau |
| Sarah Schulman | *After Delores* | 1988 | US | Cau |
| Sarah Schulman | *Empathy* | 1992 | US | Cau |
| Sarah Schulman | *Rat Bohemia* | 1995 | US | Cau |
| Sarah Schulman | *The Mere Future* | 2009 | US | Cau |
| Jewelle Gomez | *The Gilda Stories* | 1991 | US | Bla |
| Karin Kallmaker | *Touchwood* | 1991 | US | Cau |
| Karin Kallmaker | *Paperback Romance* | 1992 | US | Cau |
| Karin Kallmaker | *Painted Moon* | 1994 | US | Cau |
| Karin Kallmaker | *Wild Things* | 1996 | US | Cau |
| Karin Kallmaker | *Embrace in Motion* | 1997 | US | Cau |
| Karin Kallmaker | *Christabel* | 1998 | US | Cau |
| Karin Kallmaker | *Unforgettable* | 2000 | US | Cau |
| Karin Kallmaker | *Maybe Next Time* | 2003 | US | Cau |
| Karin Kallmaker | *One Degree of Separation* | 2004 | US | Cau |
| Karin Kallmaker | *Just Like That* | 2005 | US | Cau |
| Karin Kallmaker | *Finders Keepers* | 2006 | US | Cau |
| Karin Kallmaker | *Warming Trend* | 2009 | US | Cau |
| Karin Kallmaker | *Stepping Stone* | 2009 | US | Cau |
| Karin Kallmaker | *Above Temptation* | 2010 | US | Cau |
| Karin Kallmaker | *Comfort and Joy* | 2012 | US | Cau |
| Karin Kallmaker | *Love by the Numbers* | 2013 | US | Cau |
| Karin Kallmaker | *Captain of Industry* | 2016 | US | Cau |
| Stella Duffy | *Calendar Girl* | 1994 | ENG | Cau |
| Stella Duffy | *Wavewalker* | 1996 | ENG | Cau |
| Stella Duffy | *Beneath the Blonde* | 1997 | ENG | Cau |
| Stella Duffy | *Fresh Flesh* | 1999 | ENG | Cau |
| Stella Duffy | *Mouths of Babes* | 2005 | ENG | Cau |
| Nina Revoyr | *The Necessary Hunger* | 1997 | US | Mix |
| Nina Revoyr | *The Age of Dreaming* | 2008 | US | Mix |
| Nina Revoyr | *Wingshooters* | 2011 | US | Mix |
| Shamim Sarif | *The World Unseen* | 2001 | ENG | Mix |
| Shamim Sarif | *Despite the Falling Snow* | 2004 | ENG | Mix |
| Shamim Sarif | *I Can't Think Straight* | 2008 | ENG | Mix |
| Ali Smith | *Hotel World* | 2001 | SCT | Cau |
| Ali Smith | *The Accidental* | 2005 | SCT | Cau |
| Ali Smith | *Girl Meets Boy* | 2007 | SCT | Cau |
| Ali Smith | *There But For The* | 2011 | SCT | Cau |
| Ali Smith | *Artful* | 2012 | SCT | Cau |
| Ali Smith | *How to Be Both* | 2014 | SCT | Cau |
| Ali Smith | *Winter* | 2017 | SCT | Cau |
| Karen X. Tulchinsky | *The Five Books of Moses Lapinsky* | 2003 | CAN | Cau |
| Karin Slaughter | *Indelible* | 2004 | US | Cau |
| Karin Slaughter | *Faithless* | 2005 | US | Cau |
| Karin Slaughter | *Triptych* | 2006 | US | Cau |
| Karin Slaughter | *Fractured* | 2008 | US | Cau |
| Karin Slaughter | *Broken* | 2010 | US | Cau |
| Karin Slaughter | *Criminal* | 2012 | US | Cau |
| Karin Slaughter | *Cop Town* | 2014 | US | Cau |
| Karin Slaughter | *The Kept Woman* | 2016 | US | Cau |
| Valerie Mason-John | *Borrowed Body* | 2005 | ENG | Bla |
| Ellis Avery | *The Teahouse Fire* | 2006 | US | Cau |
| Joanna Briscoe | *Sleep With Me* | 2005 | ENG | Cau |
| Joanna Briscoe | *You* | 2011 | ENG | Cau |
| Ellen Hart | *Night Vision* | 2006 | US | Cau |
| Ellen Hart | *Sweet Poison* | 2008 | US | Cau |
| Nicola Griffith | *Always* | 2007 | ENG | Cau |
| Nicola Griffith | *So Lucky* | 2018 | ENG | Cau |
| Helen Humphreys | *The Frozen Thames* | 2007 | CAN | Cau |
| Helen Humphreys | *The Reinvention of Love* | 2011 | CAN | Cau |
| Helen Humphreys | *The Evening Chorus* | 2015 | CAN | Cau |
| Emma Donoghue | *The Sealed Letter* | 2008 | IRL/CAN | Cau |
| Emma Donoghue | *Room* | 2010 | IRL/CAN | Cau |
| Emma Donoghue | *Frog Music* | 2014 | IRL/CAN | Cau |
| Emma Donoghue | *The Wonder* | 2016 | IRL/CAN | Cau |
| Cheryl Rainfield | *Scars* | 2010 | CAN | Cau |
| Cheryl Rainfield | *Stained* | 2013 | CAN | Cau |
| Jessie Chandler | *Bingo Barge Murder* | 2011 | US | Cau |
| Jessie Chandler | *Hide and Snake Murder* | 2012 | US | Cau |
| Jessie Chandler | *Pickle in the Middle Murder* | 2013 | US | Cau |
| Jessie Chandler | *Chip Off the Old Ice Block* | 2014 | US | Cau |
| B. K. Loren | *Theft* | 2012 | US | Cau |
| Charlotte Mendelson | *Almost English* | 2013 | ENG | Cau |
| Laura Antoniou | *The Killer Wore Leather* | 2013 | US | Cau |
| Melissa Scott | *Ouroboros* | 2013 | US | Cau |
| Melissa Scott | *Fairs' Point* | 2014 | US | Cau |
| Melissa Scott | *Point of Sighs* | 2018 | US | Cau |
| Tamai Kobayashi | *Prairie Ostrich* | 2014 | CAN | Asian |
| Judith Frank | *All I Love and Know* | 2014 | US | Cau |
| Lucy Jane Bledsoe | *A Thin Bright Line* | 2016 | US | Cau |
| Hilary Bonner | *Deadly Dance* | 2017 | ENG | Cau |
| Kristyn Dunnion | *Tarry This Night* | 2017 | CAN | Cau |
| Paula Martinac | *The Ada Decades* | 2017 | US | Cau |
| Lydia Kwa | *Oracle Bone* | 2017 | CAN | Asian |
| Amber Dawn | *Sodom Road Exit* | 2018 | CAN | Cau |
| Blanche McCrary Boyd | *Tomb of the Unknown Racist* | 2018 | US | Cau |

## Table S6. List of bisexual female novelists

| Author | Novel | Publication year | Country | Race |
| --- | --- | --- | --- | --- |
| Aphra Behn | *The Fair Jilt* | 1688 | ENG | Cau |
| Aphra Behn | *Oroonoko* | 1688 | ENG | Cau |
| Aphra Behn | *The History of the Nun: or, the Fair Vow-Breaker* | 1689 | ENG | Cau |
| Mary Eleanor Wilkins Freeman | *Jane Field* | 1892 | US | Cau |
| Mary Eleanor Wilkins Freeman | *Pembroke* | 1894 | US | Cau |
| Mary Eleanor Wilkins Freeman | *Madelon* | 1896 | US | Cau |
| Mary Eleanor Wilkins Freeman | *Jerome, A Poor Man* | 1897 | US | Cau |
| Mary Eleanor Wilkins Freeman | *The Jamesons* | 1899 | US | Cau |
| Mary Eleanor Wilkins Freeman | *Evelina's Garden* | 1899 | US | Cau |
| Mary Eleanor Wilkins Freeman | *The Heart's Highway: A Romance of Virginia in the Seventeenth Century* | 1900 | US | Cau |
| Mary Eleanor Wilkins Freeman | *The Portion of Labor* | 1901 | US | Cau |
| Mary Eleanor Wilkins Freeman | *The Debtor* | 1905 | US | Cau |
| Mary Eleanor Wilkins Freeman | *By the Light of the Soul* | 1907 | US | Cau |
| Mary Eleanor Wilkins Freeman | *The Shoulders of Atlas* | 1908 | US | Cau |
| Mary Eleanor Wilkins Freeman | *The Butterfly House* | 1912 | US | Cau |
| Mary MacLane | *I Await the Devil's Coming* | 1902 | CAN/US | Cau |
| Henry Handel Richardson | *Maurice Guest* | 1908 | AUS | Cau |
| Henry Handel Richardson | *The Getting of Wisdom* | 1910 | AUS | Cau |
| Henry Handel Richardson | *Australia Felix* | 1917 | AUS | Cau |
| Henry Handel Richardson | *The Way Home* | 1925 | AUS | Cau |
| Henry Handel Richardson | *Ultima Thule* | 1929 | AUS | Cau |
| Vita Sackville-West | *The Heir* | 1922 | ENG | Cau |
| Vita Sackville-West | *Challenge* | 1923 | ENG | Cau |
| Vita Sackville-West | *Seducers in Ecuador* | 1924 | ENG | Cau |
| Vita Sackville-West | *Passenger to Teheran* | 1926 | ENG | Cau |
| Vita Sackville-West | *All Passion Spent* | 1931 | ENG | Cau |
| Vita Sackville-West | *Family History* | 1932 | ENG | Cau |
| Elizabeth Bowen | *The Hotel* | 1927 | IRL/ENG | Cau |
| Elizabeth Bowen | *The Last September* | 1929 | IRL/ENG | Cau |
| Elizabeth Bowen | *Friends and Relations* | 1931 | IRL/ENG | Cau |
| Elizabeth Bowen | *To the North* | 1932 | IRL/ENG | Cau |
| Elizabeth Bowen | *The Death of the Heart* | 1938 | IRL/ENG | Cau |
| Elizabeth Bowen | *The Heat of the Day* | 1949 | IRL/ENG | Cau |
| Elizabeth Bowen | *A World of Love* | 1955 | IRL/ENG | Cau |
| Elizabeth Bowen | *The Little Girls* | 1964 | IRL/ENG | Cau |
| Djuna Barnes | *Nightwood* | 1936 | US | Cau |
| Carson McCullers | *The Heart Is a Lonely Hunter* | 1940 | US | Cau |
| Carson McCullers | *The Member of the Wedding* | 1946 | US | Cau |
| Jane Bowles | *Two Serious Ladies* | 1943 | US | Cau |
| Anaïs Nin | *A Spy In the House of Love* | 1954 | CU-US | Cau |
| Iris Murdoch | *Under the Net* | 1954 | IRL/ENG | Cau |
| Iris Murdoch | *The Unicorn* | 1963 | IRL/ENG | Cau |
| Iris Murdoch | *The Italian Girl* | 1964 | IRL/ENG | Cau |
| Iris Murdoch | *The Time of the Angels* | 1966 | IRL/ENG | Cau |
| Iris Murdoch | *Bruno's Dream* | 1969 | IRL/ENG | Cau |
| Iris Murdoch | *A Word Child* | 1975 | IRL/ENG | Cau |
| Iris Murdoch | *The Sea, the Sea* | 1978 | IRL/ENG | Cau |
| Valerie Taylor | *Whisper Their Love* | 1957 | US | Cau |
| Valerie Taylor | *Unlike Others* | 1963 | US | Cau |
| Valerie Taylor | *Return to Lesbos* | 1963 | US | Cau |
| Doris Grumbach | *Chamber Music* | 1979 | US | Cau |
| Doris Grumbach | *The Missing Person* | 1981 | US | Cau |
| Doris Grumbach | *The Ladies* | 1984 | US | Cau |
| Doris Grumbach | *The Magician’s Girl* | 1987 | US | Cau |
| Doris Grumbach | *The Book of Knowledge* | 1995 | US | Cau |
| Kathy Acker | *Blood and Guts in High School* | 1984 | US | Cau |
| Susan Sontag | *The Volcano Lover* | 1992 | US | Cau |
| Elizabeth Gilbert | *Stern Men* | 2000 | US | Cau |
| Elizabeth Gilbert | *The Signature of All Things* | 2013 | US | Cau |
| Sara Ryan | *Empress of the World* | 2001 | US | Cau |
| Ann Herendeen | *Pride/Prejudice* | 2010 | US | Cau |
| Sapphire | *The Kid* | 2011 | US | Bla |
| Andrea Goldsmith | *The Memory Trap* | 2013 | AUS | Cau |
| Cynthia Bond | *Ruby* | 2014 | US | Bla |
| Catherine Hernandez | *Scarborough* | 2017 | CAN | Mix |

## Table S7. Descriptive statistics of the study sample

|  | Heterosexual males  *M SD* | | Heterosexual females  *M SD* | | Homosexual males  *M SD* | | Homosexual females  *M SD* | | Bisexual females  *M SD* | |
| --- | --- | --- | --- | --- | --- | --- | --- | --- | --- | --- |
| Age | 41.17 | 8.05 | 43.00 | 8.82 | 42.43 | 11.33 | 43.85 | 10.34 | 46.00 | 12.18 |
| Publ. year | 1942 | 58.65 | 1945 | 60.92 | 1975 | 38.60 | 1985 | 32.65 | 1935 | 65.63 |
| Novels | 151 | | 153 | | 167 | | 158 | | 65 | |
| Authors | 86 | | 85 | | 55 | | 54 | | 22 | |
| Word count | 16.8 million | | 15.9 million | | 15.7 million | | 13 million | | 5.5 million | |

**Table S8.** Multilevel model, which included three predictor variables: sex, publication year, and author’s age at publication. Results based on samples of 151 male-authored (word count 16.8 million words by a total of 86 novelists) and 153 female-authored novels (word count 15.9 million words by a total of 85 novelists). All authors are heterosexual. Publication year and author’s age at publication were grand-mean centred to provide interpretable values for the intercept (Biesanz, Deeb-Sossa, Papadakis, Bollen, & Curran, 2004; Heck, Thomas, & Tabata, 2011). Cohen’s *d*s are given for sex differences, calculated using the following formula: *d* = *b* / *SD*_pooled_ (Feingold, 2015). Positive effects sizes indicate higher values in male-authored novels; negative effect sizes indicate higher values in female-authored novels.

*Category* *Estimate* *SE*  *df* *t*  *p* *95% CIs* *d 95%CId*

| **Analytic**  (Intercept)  Sex  Pub. year  Age | 62.543  8.538  –0.011  –0.004 | 1.223  1.732  0.015  0.086 | 165  167  168  298 | 51.157  4.929  –0.746  –0.046 | <.001  <.001  0.457  0.963 | 60.129  5.118  –0.040  –0.173 | 64.958  11.958  0.018  0.165 | 0.68 | 0.41 | 0.95 |
| --- | --- | --- | --- | --- | --- | --- | --- | --- | --- | --- |
| **≥6 letters**  (Intercept)  Sex  Pub. year  Age | 15.357  0.479  –0.011  0.020 | 0.227  0.322  0.003  0.015 | 166  168  168  290 | 67.604  1.490  –3.957  1.341 | <.001  0.138  <.001  0.181 | 14.908  –0.156  –0.016  –0.009 | 15.805  1.114  –0.005  0.050 | 0.20*^ns^* | –0.07 | 0.47 |
| **P. pronoun**  (Intercept)  Sex  Pub. year  Age | 11.679  –1.155  0.007  –0.026 | 0.162  0.230  0.002  0.012 | 160  163  163  300 | 72.164  –5.033  3.484  –2.189 | <.001  <.001  0.001  0.029 | 11.359  –1.609  0.003  –0.050 | 11.999  –0.702  0.011  –0.003 | –0.66 | –0.91 | –0.40 |
| **Articles**  (Intercept)  Sex  Pub. year  Age | 7.390  1.158  0.002  –0.003 | 0.111  0.156  0.001  0.007 | 164  166  166  286 | 66.849  7.399  1.394  –0.445 | <.001  <.001  0.165  0.657 | 7.172  0.849  –0.001  –0.017 | 7.608  1.467  0.005  0.011 | 1.05 | 0.77 | 1.33 |
| **Pos. emot.**  (Intercept)  Sex  Pub. year  Age | 2.799  –0.401  –0.008  0.008 | 0.053  0.075  0.001  0.003 | 164  166  166  289 | 52.746  –5.331  –12.698  2.189 | <.001  <.001  <.001  0.029 | 2.694  –0.549  –0.009  0.001 | 2.904  –0.252  –0.007  0.014 | –0.55 | –0.76 | –0.35 |
| **Neg. emot.**  (Intercept)  Sex  Pub. year  Age | 2.047  –0.103  –0.002  –0.004 | 0.038  0.054  <.001  0.003 | 162  164  165  296 | 53.214  –1.885  –4.995  –1.438 | <.001  0.061  <.001  0.152 | 1.971  –0.210  –0.003  –0.009 | 2.123  0.005  –0.001  0.001 | –0.24*^ns^* | –0.50 | 0.01 |
| **Anxiety**  (Intercept)  Sex  Pub. year  Age | 0.423  –0.043  –0.001  –0.001 | 0.011  0.015  <.001  <.001 | 166  168  168  298 | 38.934  –2.777  –4.425  –1.216 | <.001  0.006  <.001  0.225 | 0.402  –0.073  –0.001  –0.002 | 0.445  –0.012  0.000  0.001 | –0.36 | –0.62 | –0.11 |
| **Anger**  (Intercept)  Sex  Pub. year  Age | 0.457  0.066  0.001  –0.002 | 0.020  0.028  <.001  0.001 | 161  163  164  300 | 22.958  2.344  2.262  –1.231 | <.001  0.020  0.025  0.219 | 0.418  0.010  0.000  –0.005 | 0.496  0.122  0.001  0.001 | 0.32 | 0.05 | 0.58 |
| **Sad**  (Intercept)  Sex  Pub. year  Age | 0.589  –0.091  –0.002  –0.002 | 0.013  0.019  <.001  0.001 | 164  167  168  293 | 44.514  –4.842  –9.482  –1.576 | <.001  <.001  <.001  0.116 | 0.563  –0.128  –0.002  –0.004 | 0.615  –0.054  –0.001  <.001 | –0.52 | –0.73 | –0.31 |
| **Social**  (Intercept)  Sex  Pub. year  Age | 13.839  –1.319  0.003  –0.029 | 0.171  0.242  0.002  0.013 | 155  157  158  300 | 81.118  –5.455  1.513  –2.333 | <.001  <.001  0.132  0.020 | 13.502  –1.797  –0.001  –0.054 | 14.176  –0.842  0.007  –0.005 | –0.74 | –1.00 | –0.47 |
| **Cog. proc.**  (Intercept)  Sex  Pub. year  Age | 10.142  –0.745  –0.006  0.025 | 0.152  0.216  0.002  0.011 | 168  170  171  297 | 66.573  –3.453  –3.276  2.321 | <.001  0.001  0.001  0.021 | 9.841  –1.171  –0.010  0.004 | 10.443  –0.319  –0.002  0.045 | –0.47 | –0.73 | –0.20 |
| **Different.**  (Intercept)  Sex  Pub. year  Age | 2.936  –0.149  –0.002  0.014 | 0.049  0.069  <.001  0.003 | 167  169  169  280 | 60.088  –2.154  –3.806  4.733 | <.001  0.033  <.001  <.001 | 2.840  –0.285  –0.003  0.008 | 3.032  –0.012  –0.001  0.020 | –0.30 | –0.57 | –0.02 |
| **Conjunct.**  (Intercept)  Sex  Pub. year  Age | 6.505  –0.197  –0.006  0.008 | 0.084  0.118  0.001  0.006 | 170  172  173  297 | 77.888  –1.667  –6.047  1.337 | <.001  0.097  <.001  0.182 | 6.341  –0.431  –0.008  –0.004 | 6.670  0.036  –0.004  0.019 | –0.21*^ns^* | –0.46 | 0.04 |
| **Sexual**  (Intercept)  Sex  Pub. year  Age | 0.105  0.027  <.001  –0.001 | 0.012  0.017  <.001  <.001 | 174  177  177  299 | 8.867  1.611  3.189  –0.636 | <.001  0.109  0.002  0.525 | 0.082  –0.006  <.001  –0.002 | 0.128  0.060  <.001  0.001 | 0.22*^ns^* | –0.05 | 0.48 |
| **Death**  (Intercept)  Sex  Pub. year  Age | 0.218  0.047  <.001  0.001 | 0.011  0.015  <.001  <.001 | 153  156  157  293 | 20.671  3.171  0.322  1.375 | <.001  0.002  0.748  0.170 | 0.197  0.018  0.000  0.000 | 0.239  0.077  <.001  0.003 | 0.41 | 0.15 | 0.67 |
| **Verbs**  (Intercept)  Sex  Pub. year  Age | 16.556  –1.103  0.012  –0.012 | 0.193  0.274  0.002  0.014 | 165  167  168  300 | 85.562  –4.020  4.918  –0.852 | <.001  <.001  <.001  0.395 | 16.174  –1.644  0.007  –0.039 | 16.938  –0.561  0.016  0.015 | –0.52 | –0.78 | –0.27 |
| **Past**  (Intercept)  Sex  Pub. year  Age | 7.079  –0.136  0.003  –0.003 | 0.171  0.242  0.002  0.012 | 169  171  172  299 | 41.486  –0.562  1.618  –0.239 | <.001  0.575  0.107  0.811 | 6.742  –0.613  –0.001  –0.027 | 7.416  0.341  0.007  0.021 | –0.08*^ns^* | –0.35 | 0.19 |
| **Present**  (Intercept)  Sex  Pub. year  Age | 6.919  –0.666  0.007  –0.007 | 0.183  0.259  0.002  0.013 | 167  169  170  298 | 37.877  –2.574  2.938  –0.560 | <.001  0.011  0.004  0.576 | 6.558  –1.177  0.002  –0.032 | 7.279  –0.155  0.011  0.018 | –0.35 | –0.61 | –0.08 |
| **Future**  (Intercept)  Sex  Pub. year  Age | 1.129  –0.071  –0.001  0.001 | 0.022  0.031  <.001  0.001 | 159  161  161  294 | 51.167  –2.265  –4.965  0.807 | <.001  0.025  <.001  0.420 | 1.085  –0.132  –0.002  –0.002 | 1.172  –0.009  –0.001  0.004 | –0.30 | –0.56 | –0.04 |
| **Swear**  (Intercept)  Sex  Pub. year  Age | 0.079  0.075  0.001  0.000 | 0.017  0.025  <.001  0.001 | 169  171  172  299 | 4.593  3.076  4.875  –0.156 | <.001  0.002  <.001  0.876 | 0.045  0.027  0.001  –0.003 | 0.112  0.123  0.001  0.002 | 0.39 | 0.14 | 0.64 |
| **Numbers**  (Intercept)  Sex  Pub. year  Age | 1.025  0.182  0.002  –0.001 | 0.025  0.036  <.001  0.002 | 159  162  162  300 | 40.593  5.072  5.569  –0.290 | <.001  <.001  <.001  0.772 | 0.975  0.111  0.001  –0.004 | 1.075  0.252  0.002  0.003 | 0.64 | 0.39 | 0.89 |
| **Risk**  (Intercept)  Sex  Pub. year  Age | 0.451  –0.003  –0.001  0.001 | 0.009  0.013  <.001  0.001 | 163  165  166  299 | 50.544  –0.266  –5.962  2.116 | <.001  0.791  <.001  0.035 | 0.433  –0.028  –0.001  0.000 | 0.468  0.022  0.000  0.003 | –0.03*^ns^* | –0.29 | 0.22 |
| **Space**  (Intercept)  Sex  Pub. year  Age | 7.904  0.380  0.014  –0.024 | 0.102  0.145  0.001  0.006 | 163  165  166  282 | 77.115  2.615  10.886  –3.651 | <.001  0.010  <.001  <.001 | 7.702  0.093  0.011  –0.037 | 8.106  0.666  0.016  –0.011 | 0.29 | 0.07 | 0.52 |
| **Work**  (Intercept)  Sex  Pub. year  Age | 1.111  0.019  0.001  0.003 | 0.035  0.050  <.001  0.003 | 168  170  171  300 | 31.754  0.387  2.048  1.152 | <.001  0.699  0.042  0.250 | 1.042  –0.079  0.000  –0.002 | 1.180  0.117  0.002  0.008 | 0.05*^ns^* | –0.21 | 0.32 |

## Table S9. Main effects of author’s sex on language use. Results based on samples of 151 male-authored (word count 16.8 million words by a total of 86 novelists) and 153 female-authored novels (word count 15.9 million words by a total of 85 novelists). All authors are heterosexual. Means and standard deviations are shown for males and females. Male and female means are bolded. All means and standard deviations are percentages except for analytical thinking, which is a factor-analytically derived composite score, and except for logarithm transformed values. A positive effect size indicates higher values in male-authored novels; a negative effect size indicates higher values in female-authored novels. * p < .05. ** p < .01. *** p < .001. ^ns^ = not significant. Effect sizes shown for a parametric t-test, non-parametric Whitney-Mann U test, and a multilevel model with sex, author’s age at publication, and publication year as predictor variables (MLM).

|  | Male  *M SD* | | Female  *M SD* | | Effect size (*d*)  *t* test *U* test MLM | | |
| --- | --- | --- | --- | --- | --- | --- | --- |
| Analytical | **71.26** | 12.91 | **62.42** | 12.20 | 0.70^***^ | 0.75^***^ | 0.68^***^ |
| ≥6 letters | **15.84** | 2.53 | **15.33** | 2.18 | 0.22*^ns^* | 0.26^*^ | 0.20*^ns^* |
| PPron | **10.52** | 1.88 | **11.68** | 1.63 | –0.66^***^ | –0.64^***^ | –0.66^***^ |
| PP (log_e_) | **2.34** | 0.18 | **2.45** | 0.14 | –0.68^***^ | –0.64^***^ | –0.68^***^ |
| Articles | **8.57** | 1.15 | **7.41** | 1.06 | 1.05^***^ | 1.07^***^ | 1.05^***^ |
| Pos. emot. | **2.40** | 0.62 | **2.80** | 0.82 | –0.55^***^ | –0.49^***^ | –0.55^***^ |
| PE (log_e_) | **0.84** | 0.28 | **0.99** | 0.30 | –0.50^***^ | –0.49^***^ | –0.50^***^ |
| Neg. emot. | **1.94** | 0.40 | **2.04** | 0.45 | –0.22*^ns^* | –0.23^*^ | –0.24*^ns^* |
| NE (log_e_) | **0.64** | 0.21 | **0.69** | 0.23 | –0.20*^ns^* | –0.23^*^ | –0.22*^ns^* |
| Anxiety | **0.38** | 0.12 | **0.42** | 0.12 | –0.33^**^ | –0.32^**^ | –0.36^**^ |
| Anxiety (log_e_) | **–1.01** | 0.32 | **–0.91** | 0.29 | –0.35^**^ | –0.32^**^ | –0.38^**^ |
| Anger | **0.52** | 0.26 | **0.46** | 0.15 | 0.30^**^ | 0.26^*^ | 0.32^*^ |
| Anger (log_e_) | **–0.73** | 0.37 | **–0.84** | 0.32 | 0.29^*^ | 0.26^*^ | 0.31^*^ |
| Sad | **0.50** | 0.14 | **0.59** | 0.20 | –0.48^***^ | –0.45^***^ | –0.52^***^ |
| Social | **12.52** | 1.97 | **13.80** | 1.60 | –0.71^***^ | –0.72^***^ | –0.74^***^ |
| Cog. proc. | **9.38** | 1.57 | **10.16** | 1.63 | –0.48^***^ | –0.49^***^ | –0.47^**^ |
| Differ. | **2.78** | 0.51 | **2.95** | 0.50 | –0.33^**^ | –0.39^**^ | –0.30^*^ |
| Conjunctions | **6.32** | 0.90 | **6.50** | 0.96 | –0.20*^ns^* | –0.20*^ns^* | –0.21*^ns^* |
| Sexual | **0.13** | 0.16 | **0.11** | 0.07 | 0.21*^ns^* | 0.19*^ns^* | 0.22*^ns^* |
| Death | **0.26** | 0.13 | **0.22** | 0.10 | 0.38^**^ | 0.35^**^ | 0.41^**^ |
| Verbs | **15.43** | 2.34 | **16.58** | 1.85 | –0.55^***^ | –0.59^***^ | –0.52^***^ |
| Past | **6.92** | 1.78 | **7.06** | 1.76 | –0.08*^ns^* | –0.10*^ns^* | –0.08*^ns^* |
| Present | **6.26** | 1.81 | **6.96** | 2.02 | –0.37^**^ | –0.37^**^ | –0.35^*^ |
| Present(log_e_) | **1.79** | 0.28 | **1.90** | 0.27 | –0.40^**^ | –0.37^**^ | –0.38^**^ |
| Future | **1.06** | 0.23 | **1.13** | 0.24 | –0.30^**^ | –0.32^**^ | –0.30^*^ |
| Future (log_e_) | **0.03** | 0.21 | **0.10** | 0.21 | –0.31^**^ | –0.32^**^ | –0.31^*^ |
| Swear | **0.15** | 0.26 | **0.08** | 0.09 | 0.37^**^ | 0.44^***^ | 0.39^**^ |
| Swear (log_e_)^1^ | **0.12** | 0.16 | **0.07** | 0.08 | 0.41^**^ | 0.44^***^ | 0.43^**^ |
| Numbers | **1.21** | 0.33 | **1.03** | 0.23 | 0.63^***^ | 0.61^***^ | 0.64^***^ |
| Numbers(log_e_) | **0.15** | 0.26 | **0.003** | 0.22 | 0.62^***^ | 0.61^***^ | 0.63^***^ |
| Risk | **0.45** | 0.10 | **0.45** | 0.10 | –0.06*^ns^* | –0.05*^ns^* | –0.03*^ns^* |
| Space | **8.30** | 1.29 | **7.90** | 1.29 | 0.31^**^ | 0.27^*^ | 0.29^*^ |
| Space (log_e_) | **2.10** | 0.15 | **2.05** | 0.17 | 0.32^**^ | 0.27^*^ | 0.31^*^ |
| Work | **1.13** | 0.38 | **1.11** | 0.36 | 0.04*^ns^* | 0.05*^ns^* | 0.05*^ns^* |

Note: *^1^The swear (log_e_) values were transformed by adding 1 to all of the values and then extracting the logarithm transformed values—this was done because seven novels contained zero values on the ‘swear’ variable, which would have led to missing data in logarithmic transformation.*

## Table S10. Multilevel model, which included three predictor variables: sexual orientation (heterosexual/homosexual), publication year, and author’s age at publication. Results based on samples of 151 novels by heterosexual male authors (word count 16.8 million words by a total of 86 novelists) and 167 novels by homosexual male authors (word count 15.7 million words by a total of 55 novelists). Publication year and author’s age at publication were grand-mean centred to provide interpretable values for the intercept (Biesanz et al., 2004; Heck et al., 2011). Cohen’s d and CIs were calculated using the following three formulae: 1) d = b / SD_pooled_, 2) LCI_d_ = LCI_b_ / SD_pooled_, and 3) UCI_d_ = UCI_b_ / SD_pooled_ (Feingold, 2015). Positive effect sizes indicate higher values in novels by heterosexual authors; negative effect sizes indicate higher values in novels by homosexual authors. Kenward-Roger correction was applied in SPSS. ^ns^: non-significant

*Category* *Estimate* *SE*  *df* *t*  *p* *95% CI_b_* *d 95%CI_d_*

| **Analytic**  (Intercept)  Orientation  Pub. year  Age | 62.634  7.690  –0.045  0.008 | 1.560  2.077  0.019  0.068 | 128  137  147  302 | 40.161  3.702  –2.337  0.122 | <.001  <.001  0.021  0.903 | 59.548  3.582  –0.083  –0.125 | 65.720  11.798  –0.007  0.142 | 0.60 | 0.28 | 0.92 |
| --- | --- | --- | --- | --- | --- | --- | --- | --- | --- | --- |
| **≥6 letters**  (Intercept)  Orientation  Pub. year  Age | 15.964  –0.291  –0.011  0.038 | 0.278  0.371  0.003  0.012 | 118  127  138  302 | 57.357  –0.784  –3.145  3.118 | <.001  0.434  0.002  0.002 | 15.413  –1.025  –0.018  0.014 | 16.515  0.443  –0.004  0.063 | –0.13*^ns^* | –0.45 | 0.20 |
| **P. pronoun**  (Intercept)  Orientation  Pub. year  Age | 11.377  –0.638  0.013  –0.028 | 0.193  0.259  0.002  0.010 | 115  130  147  313 | 59.075  –2.464  5.272  –2.886 | <.001  0.015  <.001  0.004 | 10.995  –1.150  0.008  –0.047 | 11.758  –0.126  0.017  –0.009 | –0.37 | –0.66 | –0.07 |
| **Articles**  (Intercept)  Orientation  Pub. year  Age | 7.582  0.926  –0.002  –0.003 | 0.126  0.168  0.002  0.005 | 127  135  143  298 | 60.093  5.518  –1.533  –0.489 | <.001  <.001  0.127  0.625 | 7.332  0.594  –0.005  –0.013 | 7.831  1.258  0.001  0.008 | 0.94 | 0.60 | 1.27 |
| **Pos. emot.**  (Intercept)  Orientation  Pub. year  Age | 2.738  –0.432  –0.006  0.007 | 0.061  0.081  0.001  0.003 | 126  135  145  302 | 44.805  –5.308  –7.770  2.471 | <.001  <.001  <.001  0.014 | 2.617  –0.593  –0.007  0.001 | 2.859  –0.271  –0.004  0.012 | –0.75 | –1.03 | –0.47 |
| **Neg. emot.**  (Intercept)  Orientation  Pub. year  Age | 2.046  –0.119  –0.001  –0.003 | 0.046  0.062  0.001  0.002 | 123  131  142  302 | 44.252  –1.926  –2.305  –1.362 | <.001  0.056  0.023  0.174 | 1.954  –0.240  –0.002  –0.007 | 2.137  0.003  0.000  0.001 | –0.31*^ns^* | –0.62 | 0.01 |
| **Anxiety**  (Intercept)  Orientation  Pub. year  Age | 0.425  –0.052  –0.001  0.000 | 0.013  0.018  <.001  0.001 | 120  130  141  305 | 31.622  –2.910  –3.009  –0.706 | <.001  0.004  0.003  0.481 | 0.399  –0.088  –0.001  –0.002 | 0.452  –0.017  0.000  0.001 | –0.46 | –0.78 | –0.15 |
| **Anger**  (Intercept)  Orientation  Pub. year  Age | 0.508  0.032  0.001  –0.002 | 0.027  0.035  <.001  0.001 | 120  130  141  305 | 19.170  0.914  2.991  –1.431 | <.001  0.363  0.003  0.153 | 0.456  –0.038  0.000  –0.004 | 0.561  0.102  0.002  0.001 | 0.15*^ns^* | –0.17 | 0.47 |
| **Sad**  (Intercept)  Orientation  Pub. year  Age | 0.546  –0.065  –0.001  –0.001 | 0.018  0.024  <.001  0.001 | 128  138  150  306 | 30.249  –2.716  –5.311  –1.689 | <.001  0.007  <.001  0.092 | 0.510  –0.113  –0.002  –0.003 | 0.581  –0.018  –0.001  0.000 | –0.39 | –0.67 | –0.11 |
| **Social**  (Intercept)  Orientation  Pub. year  Age | 13.235  –0.584  0.007  –0.011 | 0.212  0.285  0.003  0.011 | 98  115  134  314 | 62.534  –2.045  2.598  –1.011 | <.001  0.043  0.010  0.313 | 12.815  –1.149  0.002  –0.033 | 13.655  –0.018  0.012  0.011 | –0.32 | –0.62 | –0.01 |
| **Cog. proc.**  (Intercept)  Orientation  Pub. year  Age | 10.538  –1.171  –0.002  0.031 | 0.177  0.236  0.002  0.008 | 122  131  142  302 | 59.576  –4.969  –0.930  3.943 | <.001  <.001  0.354  <.001 | 10.188  –1.638  –0.006  0.015 | 10.888  –0.705  0.002  0.046 | –0.81 | –1.14 | –0.49 |
| **Different.**  (Intercept)  Orientation  Pub. year  Age | 3.024  –0.262  –0.001  0.017 | 0.064  0.085  0.001  0.002 | 131  136  142  288 | 47.199  –3.084  –1.889  7.130 | <.001  0.002  0.061  <.001 | 2.898  –0.430  –0.003  0.012 | 3.151  –0.094  0.000  0.022 | –0.50 | –0.82 | –0.18 |
| **Conjunct.**  (Intercept)  Orientation  Pub. year  Age | 6.271  –0.065  –0.007  0.015 | 0.101  0.134  0.001  0.004 | 134  140  148  295 | 62.213  –0.485  –5.289  3.762 | <.001  0.628  <.001  <.001 | 6.072  –0.330  –0.009  0.007 | 6.471  0.200  –0.004  0.023 | –0.07*^ns^* | –0.37 | 0.22 |
| **Sexual**  (Intercept)  Orientation  Pub. year  Age | 0.158  –0.012  0.001  0.000 | 0.017  0.023  <.001  0.001 | 128  137  148  304 | 9.251  –0.516  4.004  –0.522 | <.001  0.606  <.001  0.602 | 0.124  –0.057  <.001  –0.002 | 0.191  0.033  0.001  0.001 | –0.08*^ns^* | –0.39 | 0.23 |
| **Death**  (Intercept)  Orientation  Pub. year  Age | 0.231  0.038  <.001  0.000 | 0.014  0.019  <.001  0.001 | 110  124  140  312 | 16.140  1.999  1.319  0.298 | <.001  0.048  0.189  0.766 | 0.202  <.001  0.000  –0.001 | 0.259  0.076  0.001  0.002 | 0.32 | 0.00 | 0.63 |
| **Verbs**  (Intercept)  Orientation  Pub. year  Age | 16.573  –0.857  0.016  –0.024 | 0.241  0.322  0.003  0.011 | 121  130  142  305 | 68.631  –2.660  5.497  –2.233 | <.001  0.009  <.001  0.026 | 16.095  –1.495  0.011  –0.046 | 17.051  –0.220  0.022  –0.003 | –0.42 | –0.73 | –0.11 |
| **Past**  (Intercept)  Orientation  Pub. year  Age | 7.431  –0.388  0.006  –0.016 | 0.209  0.278  0.003  0.009 | 125  135  146  305 | 35.639  –1.396  2.511  –1.672 | <.001  0.165  0.013  0.095 | 7.018  –0.939  0.001  –0.034 | 7.844  0.162  0.012  0.003 | –0.23*^ns^* | –0.54 | 0.09 |
| **Present**  (Intercept)  Orientation  Pub. year  Age | 6.672  –0.312  0.007  –0.006 | 0.218  0.292  0.003  0.010 | 118  130  143  309 | 30.540  –1.067  2.429  –0.595 | <.001  0.288  0.016  0.552 | 6.239  –0.890  0.001  –0.026 | 7.104  0.266  0.012  0.014 | –0.17*^ns^* | –0.48 | 0.14 |
| **Future**  (Intercept)  Orientation  Pub. year  Age | 1.103  –0.059  –0.001  0.001 | 0.025  0.034  <.001  0.001 | 111  123  137  309 | 43.592  –1.748  –2.750  0.487 | <.001  0.083  0.007  0.626 | 1.053  –0.126  –0.001  –0.002 | 1.153  0.008  0.000  0.003 | –0.28*^ns^* | –0.59 | 0.04 |
| **Swear**  (Intercept)  Orientation  Pub. year  Age | 0.117  0.058  0.001  –0.001 | 0.024  0.032  <.001  0.001 | 121  130  142  304 | 4.964  1.848  4.657  –0.927 | <.001  0.067  <.001  0.355 | 0.071  –0.004  0.001  –0.003 | 0.164  0.121  0.002  0.001 | 0.29*^ns^* | –0.02 | 0.60 |
| **Numbers**  (Intercept)  Orientation  Pub. year  Age | 1.113  0.117  0.002  –0.001 | 0.035  0.046  <.001  0.002 | 120  130  141  305 | 32.094  2.539  3.530  –0.307 | <.001  0.012  0.001  0.759 | 1.044  0.026  0.001  –0.004 | 1.181  0.209  0.002  0.003 | 0.40 | 0.09 | 0.71 |
| **Risk**  (Intercept)  Orientation  Pub. year  Age | 0.454  –0.013  –0.001  0.002 | 0.012  0.016  <.001  0.001 | 126  135  145  303 | 38.968  –0.849  –3.123  2.993 | <.001  0.397  0.002  0.003 | 0.431  –0.044  –0.001  0.001 | 0.477  0.018  0.000  0.003 | –0.14*^ns^* | –0.45 | 0.18 |
| **Space**  (Intercept)  Orientation  Pub. year  Age | 7.897  0.554  0.011  –0.031 | 0.128  0.170  0.002  0.005 | 125  131  138  290 | 61.791  3.265  6.818  –6.109 | <.001  0.001  <.001  <.001 | 7.644  0.218  0.008  –0.040 | 8.150  0.889  0.014  –0.021 | 0.50 | 0.20 | 0.80 |
| **Work**  (Intercept)  Orientation  Pub. year  Age | 1.107  0.036  0.001  0.005 | 0.045  0.061  0.001  0.002 | 122  134  147  308 | 24.455  0.591  1.325  2.347 | <.001  0.556  0.187  0.020 | 1.017  –0.084  0.000  0.001 | 1.197  0.155  0.002  0.009 | 0.09*^ns^* | –0.22 | 0.41 |

## Table S11. Multilevel model, which included three predictor variables: female authors’ sexual orientation (heterosexual/homosexual), publication year, and author’s age at publication. Results based on samples of 153 novels by heterosexual female authors (word count 15.9 million words by a total of 85 novelists) 158 novels by homosexual female authors (word count 13 million words by a total of 54 novelists). Publication year and author’s age at publication were grand-mean centred to provide interpretable values for the intercept (Biesanz et al., 2004; Heck et al., 2011). Cohen’s d and CIs calculated using the following three formulae: 1) d = b / SD_pooled_, 2) LCI_d_ = LCI_b_ / SD_pooled_, and 3) UCI_d_ = UCI_b_ / SD_pooled_ (Feingold, 2015). Positive effect sizes indicate higher values in novels by homosexual authors; negative effect sizes indicate higher values in novels by heterosexual authors. Robust standard errors are used. ^ns^: non-significant

*Category* *Estimate* *SE*  *df* *t*  *p* *95% CI_b_* *d 95%CI_d_*

| **Analytic**  (Intercept)  Pub. year  Age  Orientation | 65.362  0.026  0.054  –2.310 | 3.267  0.019  0.137  2.408 | 135  135  135  135 | 20.007  1.380  0.392  –0.959 | <.001  0.170  0.696  0.339 | 58.934  –0.011  –0.216  –7.049 | 71.791  0.064  0.323  2.429 | –0.18*^ns^* | –0.55 | 0.19 |
| --- | --- | --- | --- | --- | --- | --- | --- | --- | --- | --- |
| **≥6 letters**  (Intercept)  Pub. year  Age  Orientation | 15.163  –0.011  0.009  0.031 | 0.566  0.003  0.019  0.391 | 135  135  135  135 | 26.813  –3.142  0.471  0.080 | <.001  0.002  0.639  0.936 | 14.050  –0.017  –0.028  –0.738 | 16.276  –0.004  0.046  0.801 | 0.01*^ns^* | –0.34 | 0.37 |
| **P. pronoun**  (Intercept)  Pub. year  Age  Orientation | 11.473  0.003  –0.046  0.167 | 0.429  0.002  0.015  0.316 | 135  135  135  135 | 26.768  1.346  –3.133 0.528 | <.001  0.181  0.002  0.598 | 10.630  –0.002  –0.075  –0.454 | 12.317  0.008  –0.017  0.788 | 0.09*^ns^* | –0.26 | 0.45 |
| **Articles**  (Intercept)  Pub. year  Age  Orientation | 7.726  0.006  0.003  –0.238 | 0.270  0.002  0.010  0.197 | 135  135  135  135 | 28.667  3.847  0.293  –1.207 | <.001  <.001  0.770  0.229 | 7.196  0.003  –0.017  –0.626 | 8.257  0.009  0.023  0.150 | –0.22*^ns^* | –0.57 | 0.14 |
| **Pos. emot.**  (Intercept)  Pub. year  Age  Orientation | 2.464  –0.010  0.005  0.172 | 0.123  <.001  0.004  0.084 | 135  135  135  135 | 20.092  –11.865  1.238  2.044 | <.001  <.001  0.218  0.043 | 2.223  –0.012  –0.003  –0.006 | 2.705  –0.008  0.013  0.338 | 0.24 | –0.01 | 0.48 |
| **Neg. emot.**  (Intercept)  Pub. year  Age  Orientation | 1.815  –0.004  –0.006  0.156 | 0.114  <.001  0.004  0.083 | 135  135  135  135 | 15.978  –5.602  –1.748  1.879 | <.001  <.001  0.083  0.062 | 1.592  –0.005  –0.013  –0.007 | 2.039  –0.002  <.001  0.319 | 0.36*^ns^* | –0.02 | 0.73 |
| **Anxiety**  (Intercept)  Pub. year  Age  Orientation | 0.381  –0.001  –0.001  0.029 | 0.031  <.001  0.001  0.022 | 135  135  135  135 | 12.468  –3.912  –1.013  1.303 | <.001  <.001  0.313  0.195 | 0.320  –0.001  –0.003  –0.015 | 0.441  0.000  0.001  0.072 | 0.24*^ns^* | –0.12 | 0.60 |
| **Anger**  (Intercept)  Pub. year  Age  Orientation | 0.370  <.001  –0.002  0.086 | 0.042  <.001  0.001  0.031 | 135  135  135  135 | 8.761  0.883  –1.708  2.772 | <.001  0.379  0.090  0.006 | 0.287  0.000  –0.005  0.025 | 0.453  <.001  <.001  0.147 | 0.52 | 0.15 | 0.88 |
| **Sad**  (Intercept)  Pub. year  Age  Orientation | 0.530  –0.002  –0.001  0.017 | 0.035  <.001  <.001  0.024 | 135  135  135  135 | 15.271  –11.690  –1.016  0.707 | <.001  <.001  0.311  0.481 | 0.462  –0.002  –0.003  –0.031 | 0.598  –0.002  0.001  0.065 | 0.10*^ns^* | –0.17 | 0.37 |
| **Social**  (Intercept)  Pub. year  Age  Orientation | 13.828  –0.002  –0.027  –0.095 | 0.405  0.002  0.013  0.302 | 135  135  135  135 | 34.132  –0.971  –2.052  –0.316 | <.001  0.333  0.042  0.753 | 13.030  –0.007  –0.053  –0.689 | 14.625  0.002  –0.001  0.499 | –0.05*^ns^* | –0.39 | 0.28 |
| **Cog. proc.**  (Intercept)  Pub. year  Age  Orientation | 9.602  –0.010  0.022  0.427 | 0.426  0.002  0.017  0.305 | 135  135  135  135 | 22.543  –4.113  1.290  1.398 | <.001  <.001  0.199  0.164 | 8.765  –0.015  –0.012  –0.174 | 10.441  –0.005  0.056  1.028 | 0.26*^ns^* | –0.11 | 0.63 |
| **Different.**  (Intercept)  Pub. year  Age  Orientation | 2.863  –0.002  0.004  0.053 | 0.127  <.001  0.005  0.089 | 135  135  135  135 | 22.620  –3.048  0.891  0.597 | <.001  0.003  0.375  0.552 | 2.614  –0.004  –0.005  –0.123 | 3.112  0.000  0.014  0.229 | 0.11*^ns^* | –0.24 | 0.45 |
| **Conjunct.**  (Intercept)  Pub. year  Age  Orientation | 6.601  –0.006  0.001  –0.195 | 0.224  0.001  0.008  0.156 | 135  135  135  135 | 29.493  –4.481  0.167  –1.258 | <.001  <.001  0.867  0.211 | 6.161  –0.009  –0.014  –0.502 | 7.042  –0.003  0.017  0.111 | –0.21*^ns^* | –0.54 | 0.12 |
| **Sexual**  (Intercept)  Pub. year  Age  Orientation | 0.065  <.001  –0.002  0.043 | 0.024  <.001  <.001  0.019 | 135  135  135  135 | 2.727  2.812  –2.017  2.267 | 0.007  0.006  0.046  0.025 | 0.018  0.000  –0.003  0.006 | 0.112  <.001  <.001  0.080 | 0.42 | 0.06 | 0.79 |
| **Death**  (Intercept)  Pub. year  Age  Orientation | 0.193  <.001  <.001  0.026 | 0.026  <.001  <.001  0.019 | 135  135  135  135 | 7.358  –0.525  0.938  1.346 | <.001  0.601  0.350  0.181 | 0.142  0.000  –0.001  –0.012 | 0.245  <.001  0.003  0.063 | 0.23*^ns^* | –0.10 | 0.56 |
| **Verbs**  (Intercept)  Pub. year  Age  Orientation | 16.340  0.004  –0.001  0.290 | 0.509  0.003  0.019  0.373 | 135  135  135  135 | 32.117  1.542  –0.037  0.776 | <.001  0.125  0.970  0.439 | 15.338  –0.001  –0.037  –0.445 | 17.341  0.010  0.036  1.024 | 0.15*^ns^* | –0.23 | 0.53 |
| **Past**  (Intercept)  Pub. year  Age  Orientation | 7.155  –0.003  0.021  –0.089 | 0.485  0.003  0.015  0.345 | 135  135  135  135 | 14.754  –1.248  1.406  –0.257 | <.001  0.214  0.162  0.798 | 6.201  –0.008  –0.008  –0.767 | 8.110  0.002  0.050  0.590 | –0.05*^ns^* | –0.41 | 0.31 |
| **Present**  (Intercept)  Pub. year  Age  Orientation | 6.613  0.008  –0.023  0.392 | 0.546  0.003  0.016  0.374 | 135  135  135  135 | 12.121  2.629  –1.440  1.048 | <.001  0.010  0.152  0.296 | 5.539  0.002  –0.055  –0.344 | 7.686  0.013  0.009  1.128 | 0.19*^ns^* | –0.17 | 0.56 |
| **Future**  (Intercept)  Pub. year  Age  Orientation | 1.046  –0.002  –0.001  0.052 | 0.052  <.001  0.002  0.036 | 135  135  135  135 | 20.285  –5.296  –0.347  1.454 | <.001  <.001  0.729  0.148 | 0.945  –0.002  –0.004  –0.018 | 1.148  –0.001  0.003  0.123 | 0.22*^ns^* | –0.08 | 0.51 |
| **Swear**  (Intercept)  Pub. year  Age  Orientation | 0.061  <.001  –0.002  0.030 | 0.023  <.001  <.001  0.018 | 135  135  135  135 | 2.656  8.160  –2.154  1.680 | 0.009  <.001  0.033  0.095 | 0.016  <.001  –0.003  –0.005 | 0.107  0.001  0.000  0.065 | 0.26*^ns^* | –0.05 | 0.57 |
| **Numbers**  (Intercept)  Pub. year  Age  Orientation | 0.984  0.001  0.003  0.069 | 0.067  <.001  0.003  0.050 | 135  135  135  135 | 14.740  3.023  1.146  1.392 | <.001  0.003  0.254  0.166 | 0.853  <.001  –0.002  –0.029 | 1.116  0.002  0.009  0.167 | 0.23*^ns^* | –0.10 | 0.57 |
| **Risk**  (Intercept)  Pub. year  Age  Orientation | 0.414  –0.001  <.001  0.029 | 0.021  <.001  <.001  0.015 | 135  135  135  135 | 20.126  –4.056  0.328  1.913 | <.001  <.001  0.743  0.058 | 0.373  –0.001  –0.001  –0.001 | 0.454  0.000  0.002  0.058 | 0.30*^ns^* | –0.01 | 0.61 |
| **Space**  (Intercept)  Pub. year  Age  Orientation | 8.448  0.016  –0.011  –0.307 | 0.252  0.001  0.009  0.178 | 135  135  135  135 | 33.473  12.347  –1.344  –1.728 | <.001  <.001  0.181  0.086 | 7.952  0.013  –0.028  –0.657 | 8.945  0.018  0.005  0.043 | –0.27*^ns^* | –0.57 | 0.04 |
| **Work**  (Intercept)  Pub. year  Age  Orientation | 1.045  <.001  <.001  0.082 | 0.133  <.001  0.003  0.103 | 135  135  135  135 | 7.867  0.961  0.198  0.797 | <.001  0.338  0.843  0.427 | 0.784  –0.001  –0.007  –0.121 | 1.306  0.002  0.008  0.284 | 0.16*^ns^* | –0.24 | 0.56 |

References

Archer, J. (2009). Does sexual selection explain human sex differences in aggression? *Behavioral and Brain Sciences*, *32*(3–4), 249–311. https://doi.org/10.1017/S0140525X09990951

Archer, J. (2019). The reality and evolutionary significance of human psychological sex differences. *Biological Reviews*, *94*(4), 1381–1415. https://doi.org/10.1111/brv.12507

Argamon, S., Goulain, J.-B., Horton, R., & Olsen, M. (2009). Vive la Différence! Text Mining Gender Difference in French Literature. *DHQ: Digital Humanities Quarterly*, *3*(2), 1–11. Retrieved from http://www.digitalhumanities.org/dhq/vol/3/2/000042/000042.html

Argamon, S., Konnel, M., Pennebaker, J. W., & Schier, J. (2007). Mining the Blogosphere: Age, gender and the varieties of self-expression. *First Monday*, *12*(9). https://doi.org/10.5210/fm.v12i9.2003

Asperholm, M., Högman, N., Rafi, J., & Herlitz, A. (2019). What Did You Do Yesterday? A Meta-Analysis of Sex Differences in Episodic Memory. *Psychological Bulletin*, *145*(8), 785–821. https://doi.org/10.1037/bul0000197

Austad, S. N. (2006). Why women live longer than men: Sex differences in longevity. *Gender Medicine*, *3*(2), 79–92. https://doi.org/10.1016/S1550-8579(06)80198-1

Austad, S. N., & Fischer, K. E. (2016). Sex Differences in Lifespan. *Cell Metabolism*, *23*(6), 1022–1033. https://doi.org/10.1016/j.cmet.2016.05.019

Baguley, T. (2009). Standardized or simple effect size: What should be reported? *British Journal of Psychology*, *100*(3), 603–617. https://doi.org/10.1348/000712608X377117

Baron-Cohen, S., Knickmeyer, R. C., & Belmonte, M. K. (2005). Sex differences in the brain: Implications for explaining autism. *Science*, Vol. 310, pp. 819–823. https://doi.org/10.1126/science.1115455

Becker, L. A. (2000). Effect Size Calculators. Retrieved from University of Colorado website: https://www.uccs.edu/~lbecker/

Biesanz, J. C., Deeb-Sossa, N., Papadakis, A. A., Bollen, K. A., & Curran, P. J. (2004). The Role of Coding Time in Estimating and Interpreting Growth Curve Models. *Psychological Methods*. https://doi.org/10.1037/1082-989X.9.1.30

Carroll, J. (2018). Minds and meaning in fictional narratives: An evolutionary perspective. *Review of General Psychology*, *22*(2), 135–146. https://doi.org/10.1037/gpr0000104

Darwin, C. (1859). *On the Origin of Species*. London: Murray.

Darwin, C. (1871). *The Descent of Man and Selection in Relation to Sex* (Vol. 1). https://doi.org/10.1017/CBO9780511703829

Del Giudice, M. (2019). Measuring sex differences and similarities. In D. P. VanderLaan & W. I. Wong (Eds.), *Gender and Sexuality Development: Contemporary Theory and Research*. New York, NY: Springer.

Feingold, A. (2015). Confidence interval estimation for standardized effect sizes in multilevel and latent growth modeling. *Journal of Consulting and Clinical Psychology*, *83*(1), 157–168. https://doi.org/10.1037/a0037721

Garson, G. (2014). *Hierarchical Linear Modeling: Guide and Applications*. https://doi.org/10.4135/9781483384450

Gottschall, J. (2008). *The Rape of Troy: Evolution, Violence, and the World of Homer*. Cambridge University Press.

Groen, Y., Fuermaier, A. B. M., Tucha, L. I., Koerts, J., & Tucha, O. (2018). How predictive are sex and empathizing–systemizing cognitive style for entry into the academic areas of social or physical sciences? *Cognitive Processing*, *19*(1), 95–106. https://doi.org/10.1007/s10339-017-0848-z

Heck, R. H., Thomas, S. L., & Tabata, L. N. (2011). *Multilevel and longitudinal modeling with IBM SPSS*. https://doi.org/10.4324/9780203855263

Hedges, L. V. (2007). Effect sizes in cluster-randomized designs. *Journal of Educational and Behavioral Statistics*, *32*(4). https://doi.org/10.3102/1076998606298043

Jockers, M. L. (2013). *Macroanalysis: Digital methods and literary history*. University of Illinois Press.

Koolen, C. (2018). *Reading beyond the female: The relationship between perception of author gender and literary quality*. Universiteit van Amsterdam.

Lai, M. H. C., & Kwok, O. M. (2016). Estimating Standardized Effect Sizes for Two- and Three-Level Partially Nested Data. *Multivariate Behavioral Research*, *51*(6), 740–756. https://doi.org/10.1080/00273171.2016.1231606

Lenhard, W., & Lenhard, A. (2016). Calculation of Effect Sizes. https://doi.org/10.13140/RG.2.2.17823.92329

Lippa, R. A. (2010a). Gender Differences in Personality and Interests: When, Where, and Why? *Social and Personality Psychology Compass*, *4*(11), 1098–1110. https://doi.org/10.1111/j.1751-9004.2010.00320.x

Lippa, R. A. (2010b). Sex differences in personality traits and gender-related occupational preferences across 53 nations: Testing evolutionary and social-environmental theories. *Archives of Sexual Behavior*, *39*(3), 619–636. https://doi.org/10.1007/s10508-008-9380-7

Luoto, S. (2019). An updated theoretical framework for human sexual selection: From ecology, genetics, and life history to extended phenotypes. *Adaptive Human Behavior and Physiology*, *5*(1), 48–102. https://doi.org/10.1007/s40750-018-0103-6

Luoto, S., Krams, I., & Rantala, M. J. (2019). A life history approach to the female sexual orientation spectrum: Evolution, development, causal mechanisms, and health. *Archives of Sexual Behavior*, *48*(5), 1273–1308. https://doi.org/10.1007/s10508-018-1261-0

Luoto, S., & van Cranenburgh, A. (2021). Psycholinguistic dataset on language use in 1145 novels published in English and Dutch. *Data in Brief*, *34*, 106655. https://doi.org/10.1016/j.dib.2020.106655

Mascaro, J. S., Rentscher, K. E., Hackett, P. D., Lori, A., Darcher, A., Rilling, J. K., & Mehl, M. R. (2018). Preliminary evidence that androgen signaling is correlated with men’s everyday language. *American Journal of Human Biology*, *30*(4), e23136. https://doi.org/10.1002/ajhb.23136

Mehl, M. R., & Pennebaker, J. W. (2003). The Sounds of Social Life: A Psychometric Analysis of Students’ Daily Social Environments and Natural Conversations. *Journal of Personality and Social Psychology*, *84*(4), 857–870. https://doi.org/10.1037/0022-3514.84.4.857

Moen, E. L., Fricano-Kugler, C. J., Luikart, B. W., & O’Malley, A. J. (2016). Analyzing clustered data: Why and how to account for multiple observations nested within a study participant? *PLoS ONE*, *11*(1), 1–17. https://doi.org/10.1371/journal.pone.0146721

Muthukrishna, M., Doebeli, M., Chudek, M., & Henrich, J. (2018). The Cultural Brain Hypothesis: How culture drives brain expansion, sociality, and life history. *PLoS Computational Biology*, *14*(11), e1006504. https://doi.org/10.1371/journal.pcbi.1006504

Nakagawa, S., & Cuthill, I. C. (2007). Effect size, confidence interval and statistical significance: A practical guide for biologists. *Biological Reviews*, *82*(4), 591–605. https://doi.org/10.1111/j.1469-185X.2007.00027.x

Nettle, D. (2007). Empathizing and systemizing: What are they, and what do they contribute to our understanding of psychological sex differences? *British Journal of Psychology*, *98*(2), 237–255. https://doi.org/10.1348/000712606X117612

Newman, M. L., Groom, C. J., Handelman, L. D., & Pennebaker, J. W. (2008). Gender differences in language use: An analysis of 14,000 text samples. *Discourse Processes*, Vol. 45, pp. 211–236. https://doi.org/10.1080/01638530802073712

Olsen, M. (2005). Écriture féminine: Searching for an indefinable practice? *Literary and Linguistics Computing*, *20*(SUPPL. 1), 147–164. https://doi.org/10.1093/llc/fqi020

Pennebaker, J. W., Chung, C. K., Frazee, J., Lavergne, G. M., & Beaver, D. I. (2014). When small words foretell academic success: The case of college admissions essays. *PLoS ONE*, *9*(12), 1–10. https://doi.org/10.1371/journal.pone.0115844

Pennebaker, James W., & Ireland, M. E. (2011). Using literature to understand authors: The case for computerized text analysis. *Scientific Study of Literature*, *1*(1), 34–48.

Rahman, Q., Abrahams, S., & Wilson, G. D. (2003). Sexual-orientation-related differences in verbal fluency. *Neuropsychology*, *17*(2), 240–246. https://doi.org/10.1037/0894-4105.17.2.240

Sjoberg, E. A., & Cole, G. G. (2018). Sex Differences on the Go/No-Go Test of Inhibition. *Archives of Sexual Behavior*, *47*(2), 537–542. https://doi.org/10.1007/s10508-017-1010-9

Su, R., Rounds, J., & Armstrong, P. I. (2009). Men and Things, Women and People: A Meta-Analysis of Sex Differences in Interests. *Psychological Bulletin*, *135*(6), 859–884. https://doi.org/10.1037/a0017364

Thelwall, M., Bailey, C., Makita, M., Sud, P., & Madalli, D. P. (2019). Gender and Research Publishing in India: Uniformly high inequality? *Journal of Informetrics*, *13*(1), 118–131. https://doi.org/10.1016/j.joi.2018.12.003

Xu, Y., Norton, S., & Rahman, Q. (2017). Sexual orientation and neurocognitive ability: A meta-analysis in men and women. *Neuroscience and Biobehavioral Reviews*, Vol. 83, pp. 691–696. https://doi.org/10.1016/j.neubiorev.2017.06.014

Zheng, L., & Zheng, Y. (2015). Sex and sexual orientation differences in empathizing-systemizing cognitive styles in China. *Personality and Individual Differences*, *87*, 267–271. https://doi.org/10.1016/j.paid.2015.08.014

1. The category of work-related words tends to focus on occupational pursuits outside the domestic sphere of child-rearing, cooking, and cleaning. To the extent that women engaged more in such domestic labour, and wrote more about it in their novels, LIWC’s overlooking domestic work-related words could cause some of the sex differences, if such differences indeed are found. It should also be noted that 19^th^ century fiction may place substantial focus on how much a character earns not by work but through possession of land, which could still be tagged as work-related word use by computerised text analysis. Though not fully inaccurate, such use of work-related words may refer to very different ways of making a livelihood than, say, those of the working class. [↑](#footnote-ref-1)
